# Beyond ferryl-mediated hydroxylation: 40 years of the rebound mechanism and C–H activation

**DOI:** 10.1007/s00775-016-1414-3

**Published:** 2016-12-01

**Authors:** Xiongyi Huang, John T. Groves

**Affiliations:** Princeton University, Princeton, USA

**Keywords:** Iron, Oxygenase, C–H activation, Rebound, Radical, Metal oxo

## Abstract

Since our initial report in 1976, the oxygen rebound mechanism has become the consensus mechanistic feature for an expanding variety of enzymatic C–H functionalization reactions and small molecule biomimetic catalysts. For both the biotransformations and models, an initial hydrogen atom abstraction from the substrate (R–**H**) by high-valent iron-oxo species (Fe^n^=O) generates a substrate radical and a reduced iron hydroxide, [Fe^n−1^–O**H ·**R]. This caged radical pair then evolves on a complicated energy landscape through a number of reaction pathways, such as oxygen rebound to form R–OH, rebound to a non-oxygen atom affording R–X, electron transfer of the incipient radical to yield a carbocation, R^+^, desaturation to form olefins, and radical cage escape. These various flavors of the rebound process, often in competition with each other, give rise to the wide range of C–H functionalization reactions performed by iron-containing oxygenases. In this review, we first recount the history of radical rebound mechanisms, their general features, and key intermediates involved. We will discuss in detail the factors that affect the behavior of the initial caged radical pair and the lifetimes of the incipient substrate radicals. Several representative examples of enzymatic C–H transformations are selected to illustrate how the behaviors of the radical pair [Fe^n−1^–O**H ·**R] determine the eventual reaction outcome. Finally, we discuss the powerful potential of “radical rebound” processes as a general paradigm for developing novel C–H functionalization reactions with synthetic, biomimetic catalysts. We envision that new chemistry will continue to arise by bridging enzymatic “radical rebound” with synthetic organic chemistry.

## Introduction

**Fig. 1:**
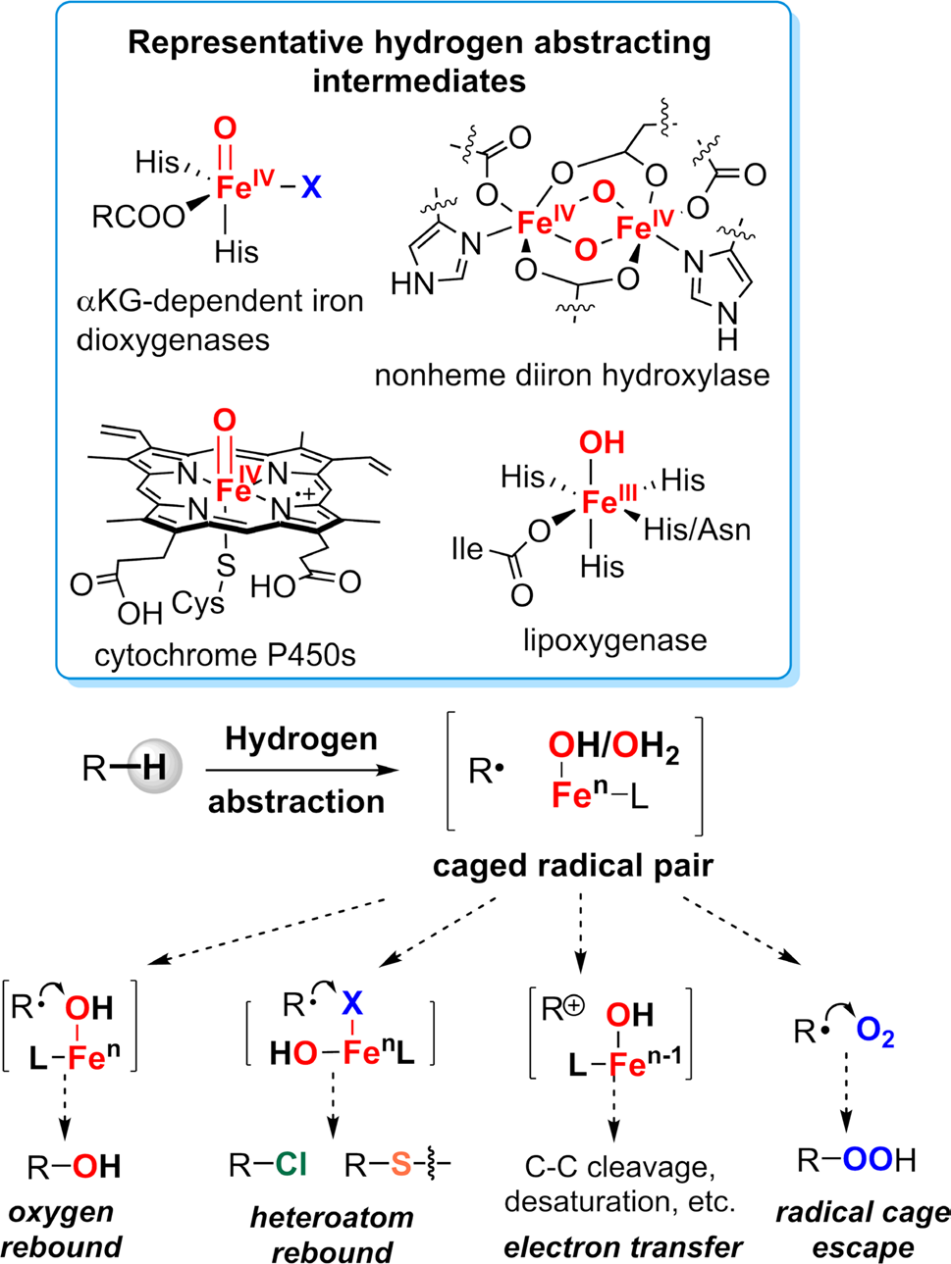
A variety of biotransformations catalyzed by iron-containing oxygenases and their mechanistic features

Molecular oxygen, with its high oxidation potential, harbors considerable energy that powers aerobic life on earth. The driving force for biological oxygenation derives ultimately from the large, 80 kcal/mol favorable enthalpic change inherent to the four-electron reduction of oxygen to water. Paradoxically, however, O_2_ in its triplet ground state is a kinetically slow oxidant due to its thermodynamic stability. The one-electron reduction of ^3^O_2_ to the superoxide ion is endergonic by 7.8 kcal/mol and the bond dissociation energy of H–OO· is only 47 kcal/mol [1–4]. Further, the triplet ground state of O_2_ imposes spin-flip barriers to two-electron processes involving closed shell reaction partners such as typical organic compounds [5, 6]. To exploit the oxidative power of O_2_, nature has developed a diverse range of enzyme cofactors to activate triplet O_2_. A good example is the family of flavin-dependent oxidases and oxygenases, in which a reduced flavin reacts readily with triplet O_2_ to form hydroperoxyflavin intermediate despite the required triplet-to-singlet spin inversion [7–9]. The majority of O_2_-activation enzymes utilize cofactors containing transition metals, such as iron, copper, and occasionally manganese. These d-block metals, with their multiple spin states and oxidation states, are employed for numerous oxidative transformations in biology such as aliphatic C–H hydroxylations and halogenations [1, 2, 10–16]. Perhaps not surprisingly, these metal-catalyzed oxidative enzymatic transformations share common mechanistic features in which oxygen is sequentially reduced to superoxo, peroxo, hydroxo, and high-valent metal-oxo intermediates, which are then exploited to activate substrates via hydrogen atom abstraction. The resulting substrate radical can then be transformed in a variety of ways (Fig. 1). Mechanistic examinations of these enzymatic transformations over the past four decades have revealed not only how these enzymes work. They have provided the impetus for the development of the field of bioinorganic chemistry, particularly high-valent metal coordination chemistry, and opened new frontiers in synthetic chemistry, led by novel, biomimetic catalytic systems for direct aliphatic C–H functionalizations [17–21].

## Mechanisms of C–H activation by cytochrome P450s and other iron-containing oxygenases

The heme-thiolate-containing monooxygenases, cytochrome P450, have assumed a uniquely important position in the hierarchy of the field and served as prototypical example to our understanding of the iron-containing oxygenases [22–25]. P450 enzymes (now termed CYP) catalyze highly selective C–H hydroxylations, as well as epoxidations, desaturations, dealkylations, and C–C bond cleavage reactions in an extremely wide range of compounds. Typical substrates include xenobiotics such as pharmaceuticals and agrochemicals and precursors for the biosynthesis of steroids, terpenoids, alkaloids, antibiotics, pigments, antioxidants, etc. Bacterial P450s have been genetically engineered for large-scale bio-transformations [26, 27].

**Fig. 2:**
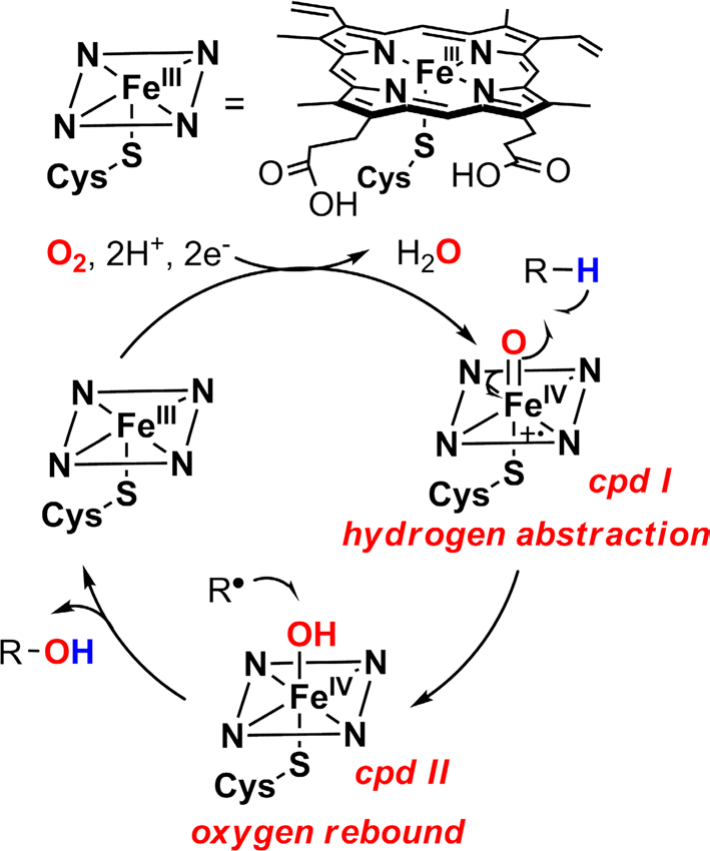
Mechanism of aliphatic C–H hydroxylation catalyzed by cytochrome P450 (the oxygen rebound mechanism)

The consensus mechanism of P450-catalyzed hydroxylation that has come to be known as the oxygen rebound mechanism is shown in Fig. 2, as proposed by us in 1970s [28–30]. The key feature of this mechanism is the involvement of an oxoiron(IV) porphyrin cation radical intermediate (compound I) that abstracts a hydrogen atom from the substrate to form [Fe^IV^–O**H ·**R] and the subsequent rebound of the incipient substrate radical to a hydroxoiron(IV) intermediate (compound II) [31, 32].

**Fig. 3:**
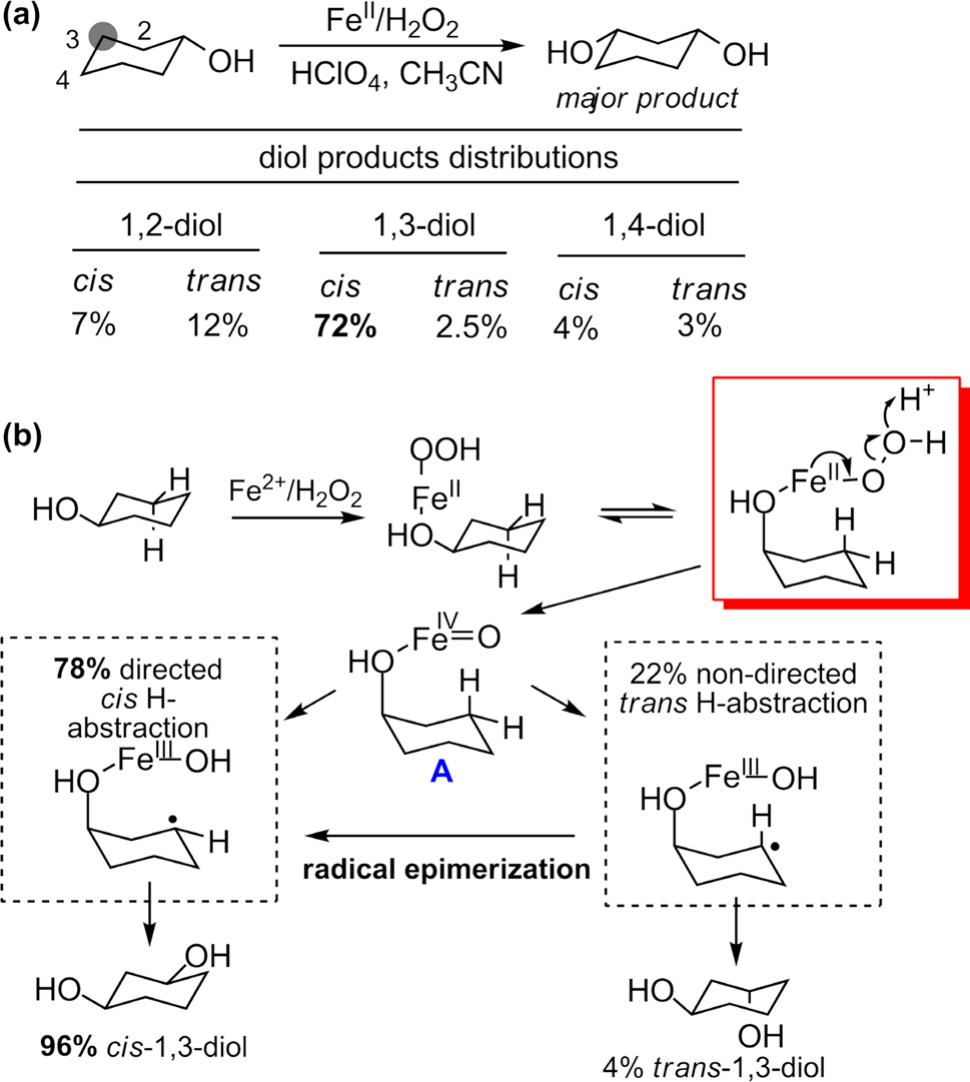
**a** Hydroxylation of cyclohexanol by Fenton’s reagent. **b** Directive effect in cyclohexanol hydroxylation by Fenton’s reagent suggests a coordinated Fe^IV^=O intermediate

The basic concept of the oxygen rebound mechanism stemmed from our early studies of Fenton’s reagent—a mixture of hydrogen peroxide and ferrous salts that can hydroxylate alkanes [33, 34]. There were extensive debates in the field at that time regarding whether the hydrogen-abstracting intermediate was a free hydroxyl radical or a high-valent iron-oxo complex [35–38]. We became interested in this topic due to intriguing common features it shared with P450s. In 1976, we reported our finding that a modified Fenton’s reagent system (Fe^2+^–H_2_O_2_–CH_3_CN) catalyzed the hydroxylation of cyclohexanol affording *cis*-1,3-cyclohexanediol as the major diol product (72%, Fig. 3a) [28]. The high regioselectivity and the apparent directive effect of the C-1 hydroxyl clearly pointed to an iron-mediated hydrogen abstraction, not hydroxyl radical. Furthermore, oxidation of *trans*-3-*trans*-5-dideuterocyclohexanol showed 78% *cis*-hydrogen abstraction at C-3 and 96% formation of *cis*-1,3-diol. This apparent two-stage directive effect indicated a stepwise hydrogen abstraction/radical oxidation sequence involving a coordinated ferryl intermediate, Fe^IV^=O (**A** in Fig. 3b). The excess *cis*-1,3-diol could be explained by a *trans*-C-3 hydrogen abstraction that then underwent radical oxidation on the *syn* side of the C-1 hydroxyl group (Fig. 3b). Today we know for sure that the two-electron oxygenation of ferrous ion by ozone produces such a ferryl species [39–43].

**Fig. 4:**
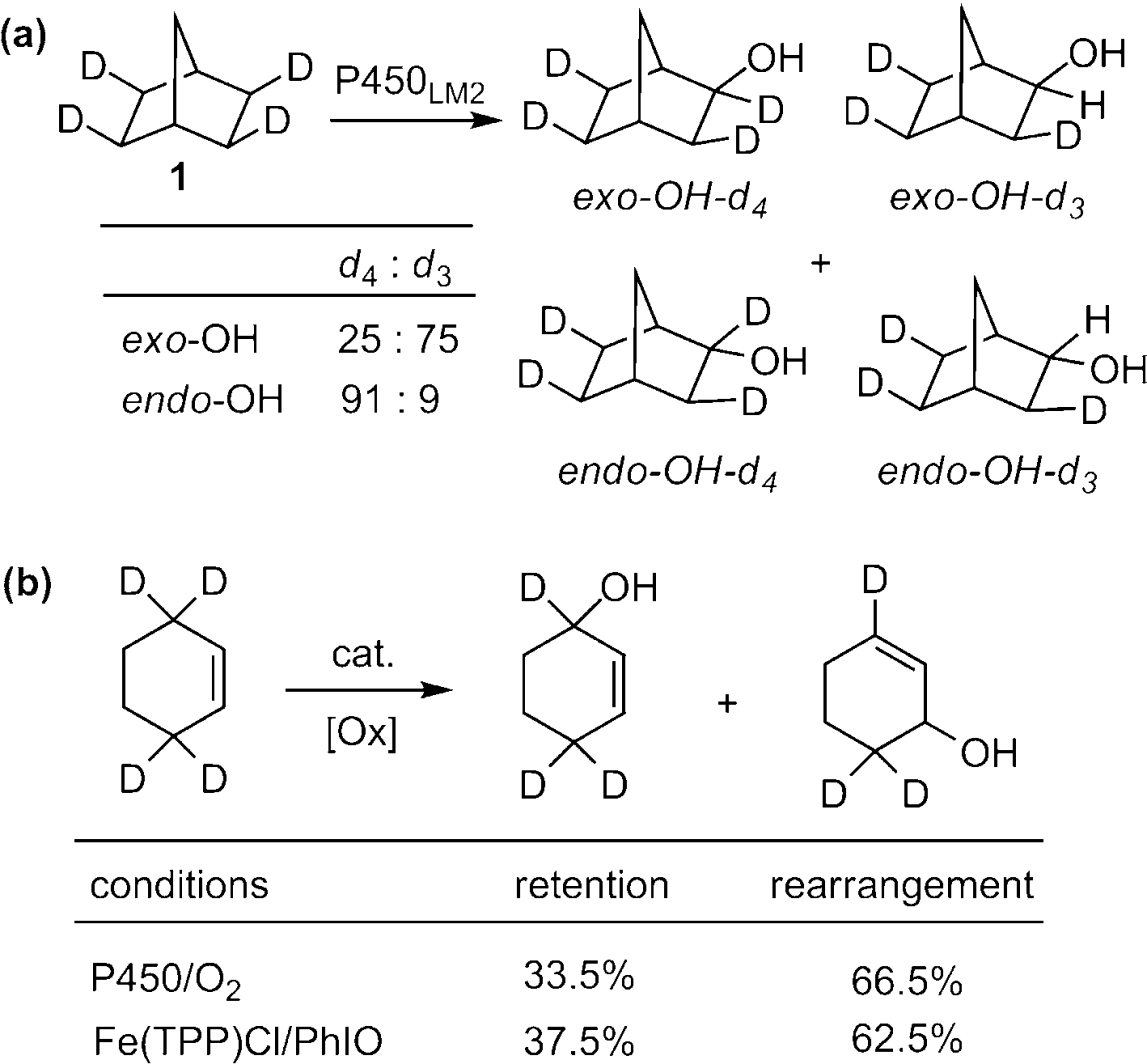
**a** Oxidation of tetradeuteronorbornane catalyzed by P450_LM2_. **b** Oxidation of 3,3,6,6-tetradeuterocyclohexene catalyzed by P450s and synthetic iron porphyrins

Closely following our studies of Fenton’s reagent, we initiated a collaboration with colleague Jud Coon to investigate the mechanism of C–H hydroxylation by rabbit liver microsomal cytochrome P450. The idea was to use selectively deuterated norbornane as a mechanistically diagnostic substrate for the enzyme. The results showed that phenobarbital-induced P450_LM2_ (now CYP2B4) catalyzed the oxidation of *exo*,*exo*,*exo*,*exo*-2,3,5,6-tetradeuteronorbornane (**1**) at the C-2 position with a significant amount of epimerization at the oxygenated carbon (Fig. 4a) [29]. We further showed that the hydroxylation of selectively deuterated cyclohexene proceeded with substantial allylic scrambling (Fig. 4b) [44]. Significantly, synthetic iron porphyrin model compounds displayed the same behavior for both norbornane and cyclohexene substrates. These discoveries provided strong evidence for a step-wise, nonconcerted mechanism for C–H scission and subsequent formation of the new C–OH bond mediated by cytochrome P450 enzymes. The presence of the intermediate substrate radical, with a lifetime long enough to epimerize and rearrange has turned out to be an important and intrinsic property of the oxygen transfer event from oxoiron complexes. A prediction of the rebound process is that the newly formed alcohol product should still be coordinated to the heme iron. Subsequently, EPR-ENDOR evidence for just such an arrangement was provided by Hoffman et al. [45].

**Fig. 5:**
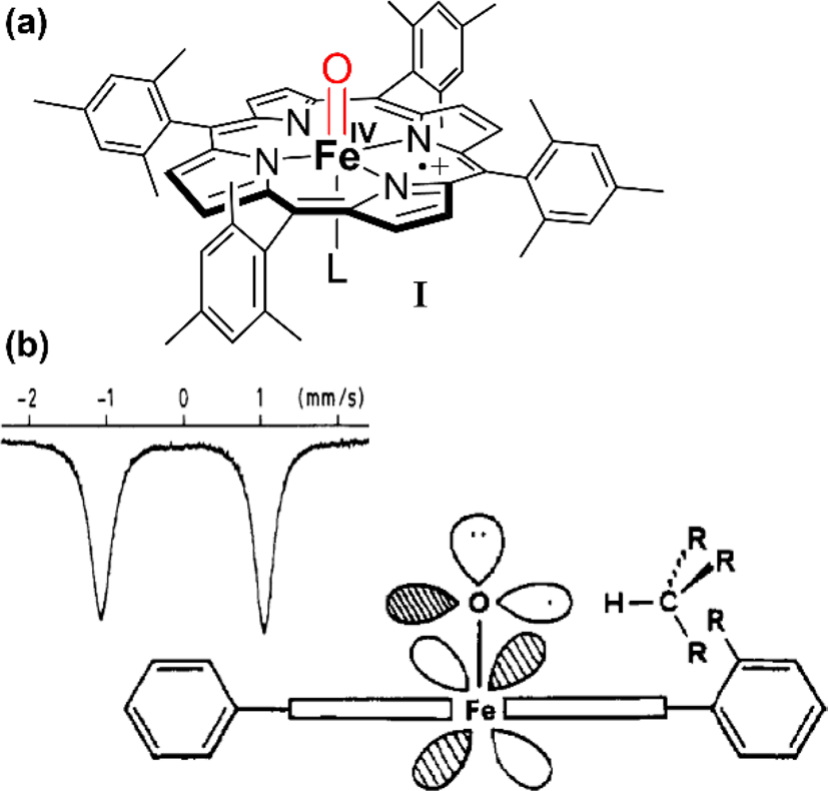
(TMP^+·^)Fe^IV^=O, the first model compound I: **a** structure of oxoiron(IV)TMP radical cation; **b** depiction of (TMP^+·^)Fe^IV^=O from reference 56 and its Mössbauer spectrum, which indicates high-valent iron

The characterization of key intermediates and analysis of their reactivity have afforded important insights into the nature of enzymatic processes. Much attention has been focused on the characterization of the hydrogen-abstracting intermediate compound I, which has been very challenging due to its highly reactive nature. The compound I of other heme-containing proteins such as horseradish peroxidase (HRP), catalase, and chloroperoxidase (CPO) are much more stable and have been well characterized as oxoiron(IV) porphyrin cation radicals [46–51]. However, these intermediates are not reactive towards unactivated hydrocarbons. Even the compound I of CPO, a heme-thiolate protein like P450s, can only slowly hydroxylate weak benzylic C–H bonds [52]. The first synthetic iron porphyrin compound I (species I in Fig. 5a) was characterized in 1981. Samples of **I** were bright green in color and displayed the characteristic broad absorbances at 406 and 645 nm in the visible spectrum of a porphyrin cation radical. The magnetic susceptibility of **I**, *μ*_B_ = 4.2, indicated an *S* = 3/2 system, while the β-pyrrole protons were observed at very high field, *δ* = −27. The Mössbauer spectrum displayed a characteristic doublet at 0.05 mm/s with quadrupolar splitting ∆*E*_q_ = 1.49 mm/s [53]. This complex, as well as HRP compound I, was shown to have a short Fe=O bond distance of 1.65 Å by EXAFS spectroscopy [54], cementing the assignment of **I** as an oxoiron(IV) porphyrin cation radical. Significantly, **I** showed high oxygen transfer reactivity towards olefins, affording epoxides even at low temperature. Notably, the generation of **I** with peroxyacids in the presence of ^18^O-water led to significant incorporation of the oxygen label into the epoxide products, indicating fast iron-oxo exchange with water: (TMP^·+^)Fe^IV^=O + H_2_^18^O ⇆ (TMP^·+^)Fe^IV^=^18^O + H_2_O. Hydroxylation of even the strong C–H bonds of alkanes was also achieved in those initial studies [55, 56]. It was observed at that time that the hydroxylation product distribution was very sensitive to the steric bulk of the iron porphyrin *meso*-substitution. This regioselectivity led us to propose that hydrogen atom abstraction from the substrate occurred through the singly occupied ferryl π* antibonding orbitals as depicted in the original, hand-drawn format (Fig. 5b).

**Fig. 6:**
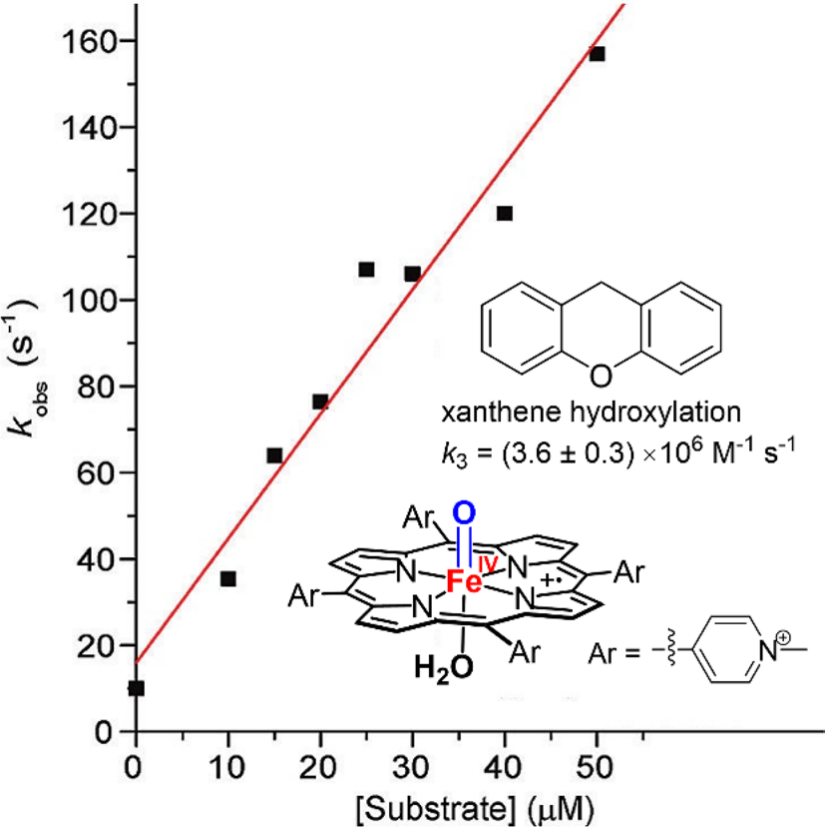
A highly reactive model ferryl porphyrin cation radical of an electron-withdrawing iron porphyrin, ref 64

Reactive high-valent metal-oxo porphyrins of manganese, chromium, and ruthenium have also been isolated and characterized [55, 57–61]. In particular, oxomanganese(V) porphyrin complexes displayed very high rates for oxygen transfer reactions in aqueous solution [62, 63]. Recently, the compound I analog of a highly electron-withdrawing, water-soluble iron porphyrin, [4-TMPyP^·+^]Fe^IV^=O, was prepared and shown to exhibit rate constants comparable to those of cytochrome P450 for benzylic C–H hydroxylation (Fig. 6) [64].

In spite of these close analogies, the lack of direct spectroscopic and kinetic characterization of a P450 compound I led to proposals of other intermediates such as iron(V)oxo and ferric hydroperoxo as alternative hydrogen-abstracting intermediates for P450s [65–67]. The long-sought P450 compound I was finally captured in 2010. Using freeze-quench techniques, Rittle and Green successfully obtained the compound I of CYP119 in high yield [68]. The near-zero chemical shift of the iron in the Mössbauer spectrum, the doublet electronic ground state signaled by the EPR spectrum, the weakened and blue-shifted Soret band in the UV, and a long-wavelength absorbance in the visible near 700 nm explicitly showed that CYP119-I is indeed an oxoiron(IV) porphyrin cation radical. These spectral signatures are reminiscent of those found in the first synthetic ferryl porphyrin cation radical (species **I**, Fig. 5) as well as the reactive [4-TMPyP^·+^]Fe^IV^=O. CYP119-I was highly reactive toward unactivated C–H bonds with an apparent rate constants in the range of 10^4^–10^7^ M^−1^ s^−1^. In 2012, a second reactive compound I was characterized by our group for the extracellular heme-thiolate aromatic peroxygenase from *Agrocybe aegerita* (*Aae*APO) [69, 70]. These newly discovered fungal peroxygenases, now called unspecific peroxygenases (UPO), are only distantly related to chloroperoxidase according to their amino acid sequences, and completely unrelated to CYP enzymes, although the proximal ligand environment, including peptide N–H hydrogen bonding to the heme-thiolate sulfur, is very similar [71]. *Aae*APO-I showed fast rate constants for substrate hydroxylations for C–H bonds up to 100 kcal/mol in the range of 10–10^5^ M^−1^ s^−1^ [69, 70], confirming compound I as the intermediate for hydrogen atom abstraction of heme-thiolate hydroxylases.

**Fig. 7:**
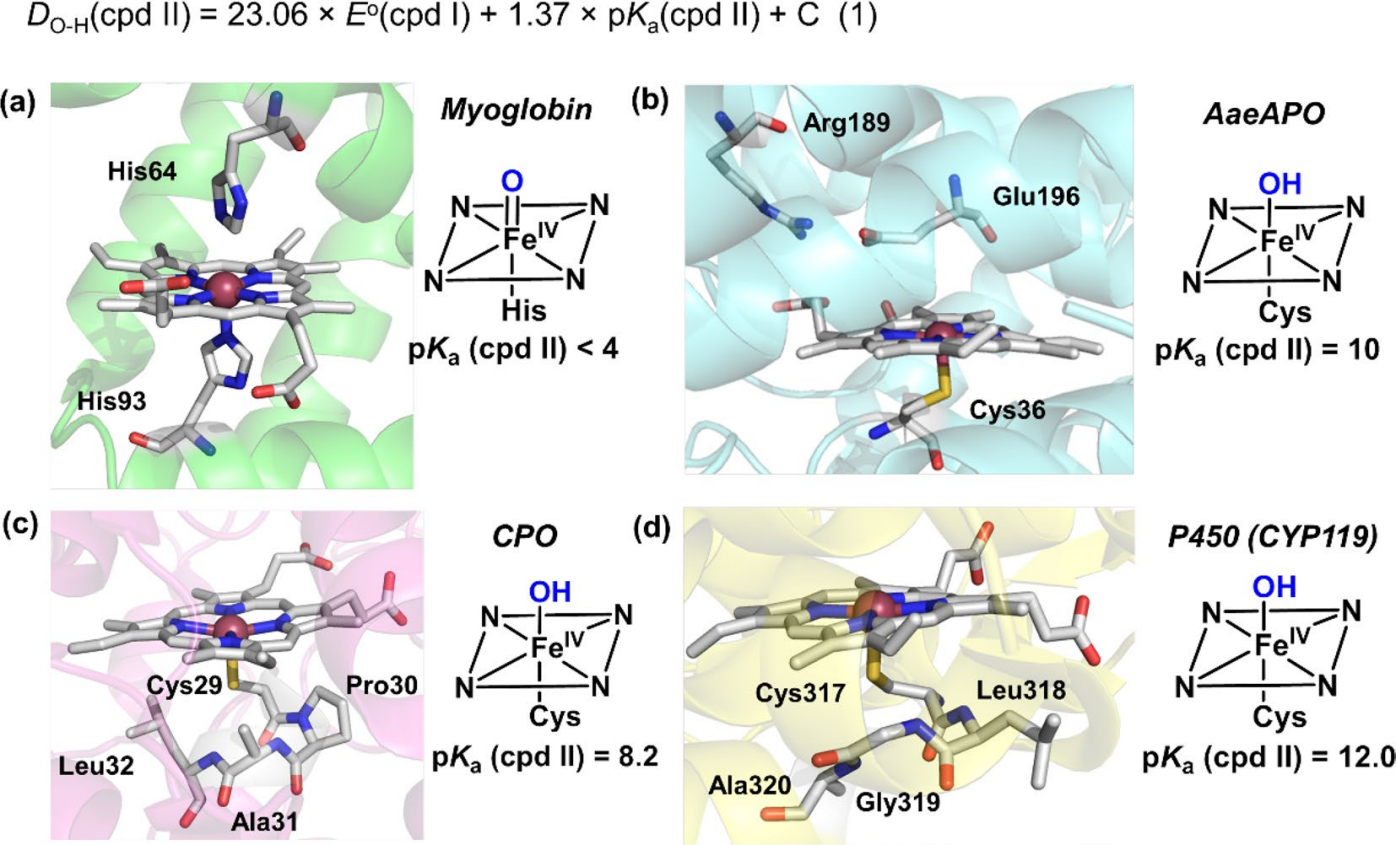
Equation 1 shows the Bordwell equation that relates the newly formed FeO–H bond energy to its p*K*_a_ and redox potential via a Hess cycle. C is a constant depending on the solvent and the electrode. For aqueous solution and normal hydrogen electrode, the value of C is 57.6 kcal/mol. Figure 2a–d shows the active site structures and p*K*_a_s of compound II for common heme-containing proteins. Active site structures were rendered using following structures: **a** myoglobin (PDB: 2V1H); **b** aromatic peroxygenase from *Agrocybe aegerita* (*Aae*APO, PDB: 2YOR); **c** chloroperoxidase (CPO, PDB: 2J19); **d** cytochrome P450 (CYP119, PDB: 1IO7). Colors: iron (*dark pink*), nitrogen (*blue*), oxygen (*red*), carbon (*silver*), sulfur (*yellow*)

Another key intermediate in P450-catalyzed hydroxylations is the hydroxoiron(IV) complex (compound II), often called the rebound intermediate [72]. The ferryl basicity of compound II that leads to the Fe^IV^O–H structure has attracted considerable interest. From insights developed by Bordwell [73, 74] and applied more recently to metal-oxo species by Mayer [75, 76], the O–H bond strength (*D*(O–H)) of compound II can be determined from the one-electron reduction potential of compound I and the p*K*_a_ of compound II using a Hess cycle (eq 1, Fig. 7). Mayer showed that the reactivity of metal-oxo systems for C–H abstraction was predominantly determined by the reaction driving force, which for the case of C–H bond scission is the free energy difference between the C–H bond that is broken and the O–H bond formed from the ferryl oxygen [77]. Accordingly, a more basic ferryl in compound II translates into a stronger Fe^IV^O–H bond, thus increasing the driving force for hydrogen atom transfer from the substrate. In this context, Green suggested that the role of the cysteine thiolate in P450 catalysis is to “push” electron density onto the ferryl oxygen, thus, making P450 compound II more basic than a typical metal-oxo species. This effect would allow for the cleavage of strong C–H bonds at biologically viable reduction potentials and reduce damage to the protein scaffold [78, 79]. Indeed, histidine-ligated heme proteins such as myoglobin and horseradish peroxidase appear to have non-basic ferryls with estimated p*K*_a_ less than 4, consistent with their slow and ineffective oxidative reactivity (Fig. 7a) [79, 80]. The heme-thiolate protein CPO is much more reactive than histidine-ligated heme proteins. The p*K*_a_ of CPO compound II was estimated to be above 8 (Fig. 7b) [81]. Very recently, Green et al. successfully determined the p*K*_a_ values of two P450s, CYP158, and CYP119 [82]. The two enzymes have similarly high compound II p*K*_a_s (around 12, Fig. 7c), although they have very different active site environments.

A highly basic compound II (p*K*_a_ = 10.0, Fig. 7d) was also observed for the heme-thiolate aromatic peroxygenase APO described above [83]. Importantly, the reduction potential of APO-I, 1.2 V with respect to the resting ferric protein, could also be determined through a Nernst equation analysis of its reversible reaction kinetics with chloride and bromide ions. This value allowed for the determination of the individual, one-electron reduction potentials of APO-I and APO-II to be *E*_cpd I/cpd II_ = 1.4 V and *E*_cpd II/ferric_ = 0.8 V at pH 7.0, and our estimate that the O–H BDE of Cys–S–Fe^IV^–O–H of APO is ∼100 kcal/mol. What this means is that for APO, and likely P450 as well, strong aliphatic C–H bonds are cleaved by these enzymes rapidly, even though there is little or no driving force for the reaction.

**Fig. 8:**
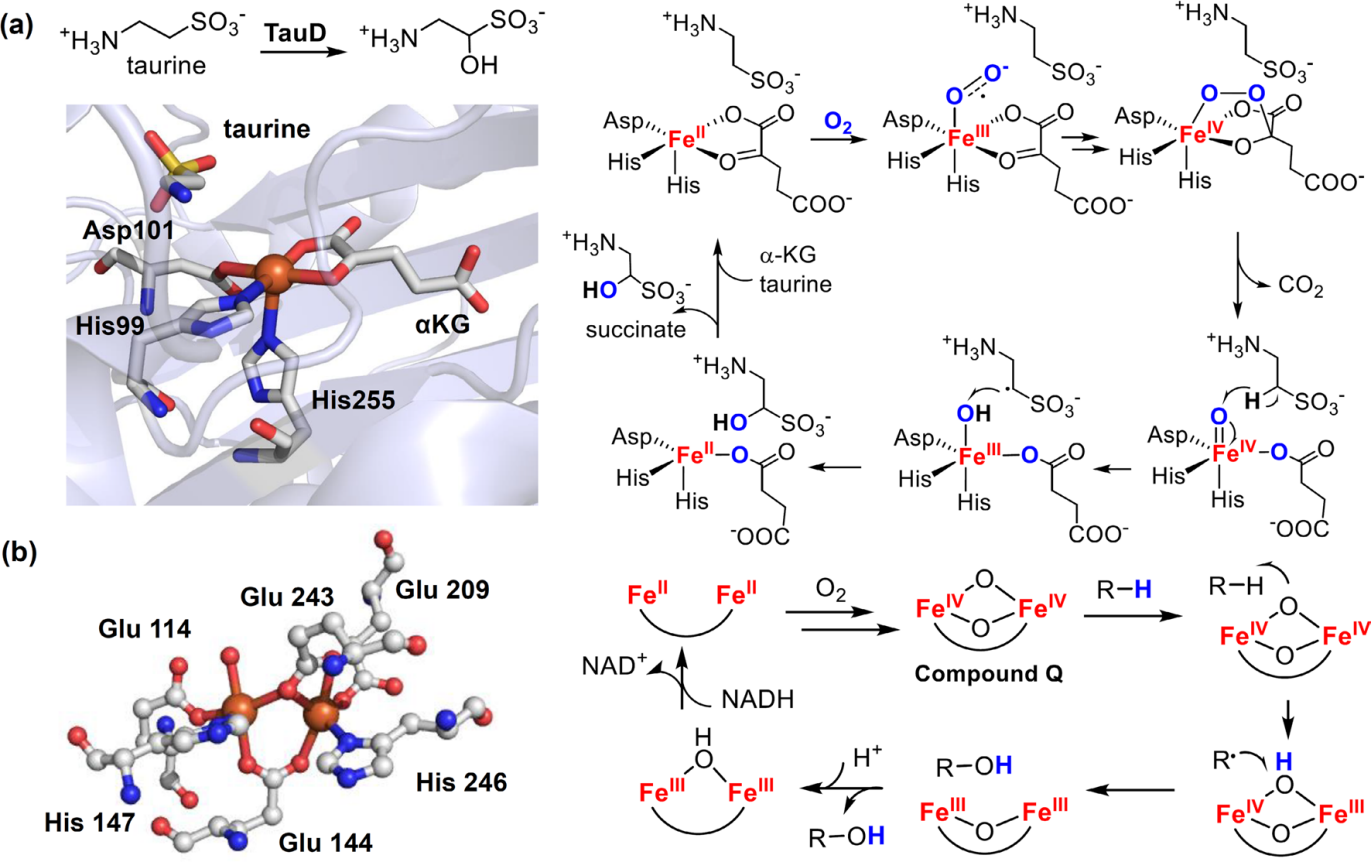
**a** Active site structure of a typical *α*KG dependent non-heme iron(II) dioxygenase, TauD, (PDB: 1OS7) and the mechanism of taurine hydroxylation catalyzed by TauD. **b** Active site structure of a representative non-heme diiron hydroxylase, soluble methane monooxygenase (sMMO) in reduced state (PDB: 1FYZ) and the typical mechanism of C–H hydroxylation catalyzed by non-heme diiron hydroxylase. Colors: iron (*orange*), oxygen (*red*), nitrogen (*blue*), sulfur (*yellow*)

Cytochrome P450 is not the only enzyme that utilizes high-valent iron-oxo intermediates to effect C–H activation reactions. Indeed, high-valent iron-oxo complexes are widely present as the reactive intermediates for the oxidative catalysis by iron-containing oxygenases [2, 5, 10]. A notable example is the superfamily of *α*-ketoglutarate (*α*KG) dependent non-heme iron(II) dioxygenases, which catalyze highly selective aliphatic C–H hydroxylations [84]. In the active site of these enzymes, the iron(II) center is coordinated facially by two histidines and one carboxylate [85, 86], a motif known as the ‘2-His-1-carboxylate facial triad’. The consensus mechanism of *α*KG-Fe(II) oxygenases is analogous to that of P450s, in which a high-valent oxoiron(IV) intermediate, first formed through O_2_ activation, abstracts a hydrogen from the substrate [87, 88]. The incipient substrate radical is then captured by a hydroxoferric intermediate, analogous to P450 compound II, to afford the hydroxylated product (Fig. 8a).

Another example of enzymes that exploit high-valent iron-oxo species for C–H activation are the non-heme diiron hydroxylases. The reactive intermediate for this family of enzymes is a bis-μ-oxo-iron(IV) complex called compound Q [89–91]. The compound Q of soluble methane monooxygenase (sMMO) has been well characterized [91–93]. Similar intermediates were also proposed for other non-heme diiron enzymes including the ω-hydroxylase AlkB, toluene monooxygenase (T4moH), and xylene monooxygenase (XylM) [89, 94]. Like compound I in P450s, compound Q initiates a hydrogen abstraction/radical rebound sequence to hydroxylate hydrocarbons, most remarkably, even methane with its 104 kcal/mol C–H bonds (Fig. 8b). From these various enzymatic reactions, it is clear that the radical rebound mechanism is a general paradigm in biological C–H oxidations catalyzed by iron-containing oxygenases, uniting the heme and non-heme enzymes.

## The intermediacy of substrate radicals during C–H activation by iron-containing oxygenases

The characteristic feature of the radical rebound mechanism is the intermediacy of the substrate radical generated in the initial hydrogen abstraction step. The properties and behavior of the incipient radical (i.e. lifetimes and conformational changes) and physical and chemical characteristics of the radical rebound step (i.e. rate constant and stereoselectivity) greatly influence the reaction outcome and are of crucial importance for the understanding of chemistries involving high-valent iron-oxo complexes. However, the transient nature of the substrate radical and the radical rebound step involving [Fe^n−1^–O**H ·**R] has generally precluded direct mechanistic studies with common kinetic and spectroscopic methods. In this regard, mechanistically diagnostic substrates, which form radicals that undergo changes in stereochemistry or structure after hydrogen abstraction, offer a powerful tool to study the intermediate radical and the rebound step [95].

**Fig. 9:**
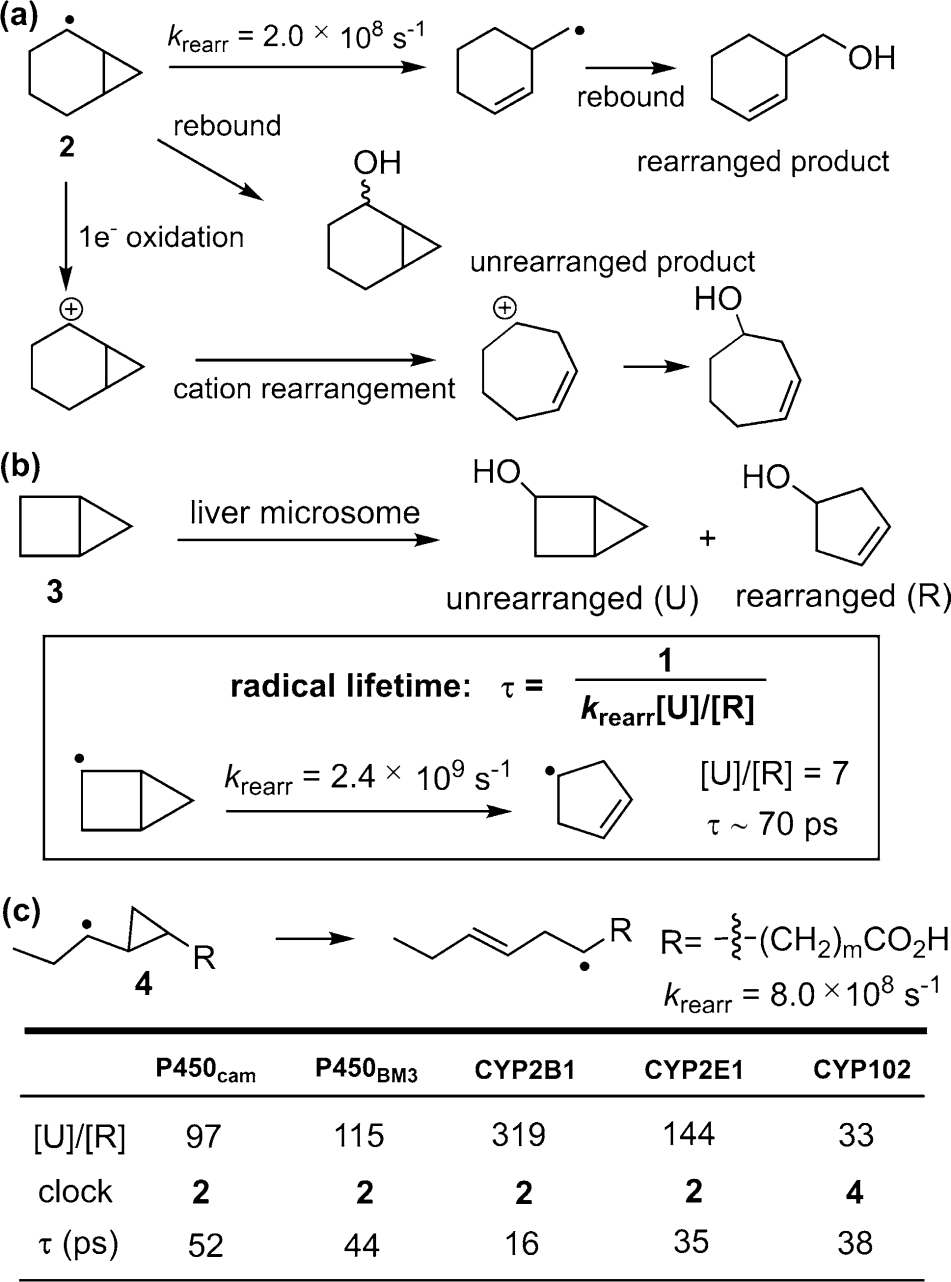
**a** Mechanisms of rearrangement of the 2-norcaranyl radical and cation intermediates. **b** Oxidation of bicyclopentane catalyzed by liver microsomal P450. **c** A representative cyclopropane-based radical clock probe and radical lifetimes of common cytochrome P450s

The first radical rearrangement studies to probe biologically relevant C–H hydroxylation used norcarane as a mechanistically diagnostic substrate and manganese porphyrins as the catalyst [55]. Significantly, the 2-norcaranyl radical (**2**) ring-opens to a primary homoallylic radical, while the corresponding cation affords the more substituted cycloheptenyl cation (Fig. 9a). The switch in reaction pathways is caused by a kinetic preference for cyclopropyl C–C bond cleavage to afford the less stable primary carbon radical, while for the cationic pathway thermodynamics prevail. Ortiz de Montellano and Stearns applied this type of substrate rearrangement probe to cytochrome P450s [96]. They found that bicyclo[2.1.0]pentane (**3**) was oxidized by rat liver microsomes containing the P450 enzyme to a mixture of ring opened and unrearranged hydroxylation products in a ratio of 1:7 (Fig. 9b). Given the known ring-opening rearrangement rate constant of 2.4 × 10^9^ s^−1^ for 2-bicyclopentanyl radical [97], a first-order rate constant of 1.4 × 10^10^ s^−1^ and an intermediate radical lifetime of ~70 ps were inferred for the oxygen rebound step. After this work, there were extensive radical clock analyses on P450s especially using norcarane [32, 98]. Norcarane is a very informative probe because the slower radical rearrangement rate of 2 × 10^8^ s^−1^ and, as discussed above, radical and cation processes afford different products (Fig. 9a) [99]. The application of norcarane to a variety of P450s gave radical rebound rates in the range between 10^10^ and 10^11^ s^−1^ corresponding to radical lifetimes also in the picosecond regime [100]. This timing is sufficient for many molecular vibrations and rotations but is too short to allow radical diffusion out of the enzyme active site. The yield of the cation-derived product cycloheptenol varied with enzymes but was generally small. A likely origin of the carbocation intermediate is an electron-transfer oxidation of the incipient carbon radical that competes with the radical oxygen rebound, as will be discussed further below. In addition to norcarane, other radical clocks such as alkyl-substituted cyclopropanes have also been employed to study the mechanism of P450-catalyzed oxidations. Rebound rate constants between 10^10^ and 10^11^ s^−1^ were generally obtained (Fig. 9c), despite some cases for which rate constants above 10^12^ s^−1^ were inferred with ‘ultrafast’ clocks based on aryl-substituted cyclopropanes [101]. Possible causes of this inconsistency include variations in the intimacy of the radical cage pair, the involvement of different spin states of the rebounding intermediate, and differences in the environment experienced by the substrate radicals within the confined space of P450 active site [1].

Similar radical-clock studies have also been performed on a variety of non-heme iron-containing enzymes that showed large variations in substrate radical lifetimes (Table 1). For instance, radical lifetimes of diiron-containing soluble methane monooxygenases (sMMO) [89, 90, 102, 103], toluene 4-monooxygenase (T4moH) [104, 105], and alkane hydroxylase alkB [106–108], were determined to be 20, 263 ps, and 1 ns, respectively. A very long radical lifetime (11 ns) was observed for monooxygenation reactions catalyzed by naphthalene dioxygenase (NDO), which belongs to the family of Rieske dioxygenases [109]. Such variations in radical lifetimes were also observed in synthetic model compounds. i.e. with the norcarane radical clock, hydroxylations catalyzed by synthetic iron porphyrins showed radical lifetimes in the order of tens to hundreds of picoseconds [110], while reactions catalyzed by manganese porphyrins exhibited radical lifetimes in the nanosecond regime [55].

**Table 1:** Typical radical lifetimes of non-heme iron enzymes and synthetic metalloporphyrins determined by norcarane

	T4moH	sMMO	AlkB	NDO	Mn(TMP)OAc	Fe-4-TMPyP
[*U*]/[*R*]	19	240	5.3	0.34	15.2	6
*τ*	263 ps	20 ps	1 ns	11 ns	330 ps	81 ps^a^

^a^Tetramethylcyclopropane (TMCP) was used as radical clock substrate

The large range of radical lifetimes for various enzymes and synthetic model compounds highlights the intricacy and inner diversity of the transient radical rebound step of [Fe^n−1^–O**H ·**R]. A fundamental question to ask is what factors control the radical lifetimes and rebound rate. From a thermodynamic perspective, radical rebound is a one-electron reduction process to the metal center. It is therefore not surprising that the metal center as well as the oxidation of the substrate radical intermediate would affect the radical rebound rate. This notion is well illustrated by the over tenfold increase in radical lifetimes for manganese porphyrin-catalyzed aliphatic hydroxylations compared to the reactions catalyzed by iron porphyrins (Table 1) [55, 110]. In sharp contrast, radical intermediates were not observed for C–H hydroxylations catalyzed by ruthenium porphyrins [110, 111]. Such variations in rebound behavior likely result from the differences in oxidation potentials, electronic configurations, as well as the relative energetics of different spin states of the rebounding intermediates [111–117].

**Fig. 10:**
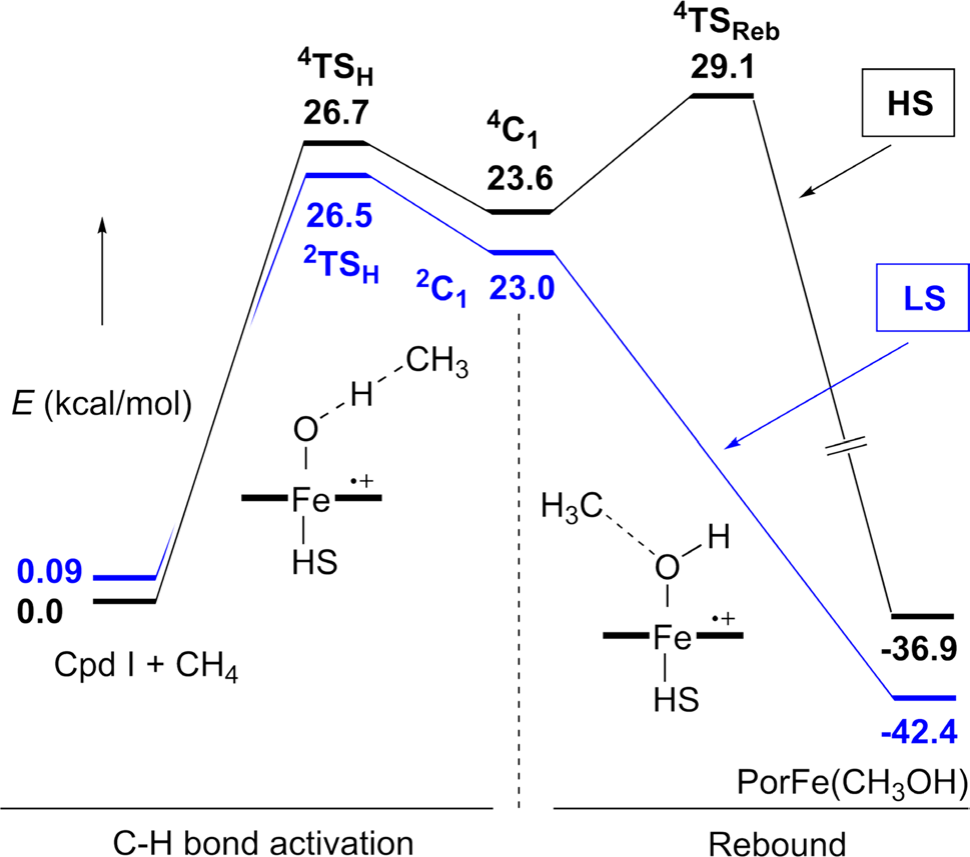
Energy profile and reaction coordinate for the methane hydroxylation by a ferryl-porphyrin cation radical. The figure is adapted from Ogliaro et al. [120]

Further insights are found by considering the frontier orbital interactions during the rebound encounter [118, 119]. Most of our understanding regarding this aspect comes from computational studies. A revealing notion gained from such studies is the possible involvement of multiple spin states and energetic surfaces for oxidations catalyzed by paramagnetic iron-oxo intermediates, which was first suggested by Shaik and his colleagues in the density functional theory (DFT) analysis of methane hydroxylation by a ferryl-porphyrin cation radical [120]. They found that the ferryl intermediate had two nearly isoenergetic electronic configurations, doublet and quartet, and thus the C–H abstraction and radical rebound could occur along either a high-spin (HS) or a low-spin (LS) trajectory (Fig. 10). Green et al. have shown that P450 compound I adopts a low spin *S* = 1/2 state [68]. Intriguingly, *S* = 3/2 spin states were generally shown for compound I of iron porphyrin model compounds [121]. These experimental findings suggest a facile interconversion between the doublet and quartet spin states of compound I. While the two pathways showed similar energy barriers for the C–H abstraction, the HS pathway exhibited a larger barrier for the radical recombination step. In both pathways, the rebound intermediate Fe^IV^–OH adopted an *S* = 1 spin state. On the LS surface, the electron of the substrate radical is predicted to interact with the singly-occupied iron *d*_xz_ (or *d*_yz_) orbital as in Fig. 5b, whereas in the HS pathway, the electron transfers into a higher-energy, unoccupied *d*_z_2 orbital, giving rise to the larger rebound barrier for rebound along the HS surface. Recently, Shaik further applied this multi-state analysis to a number of non-heme metal-oxo systems including Mn^IV^O, Fe^IV^O, Cr^IV^O, Fe^V^O, and Ru^IV^O. In these cases, the energy barriers of the rebound step were also dependent upon the spin states of the pathways [122]. The calculated rebound barriers of non-heme complexes were much higher than that of the heme intermediate in P450s. This result is consistent with the long radical lifetime measured for the non-heme iron dioxygenases and may arise from the lower oxidation state of the rebound intermediate (Fe^III^) in non-heme iron complexes.

In addition to the intrinsic properties of the rebound intermediates, another important, and often overlooked factor that affects the rebound step is the cage effect. After the initial hydrogen atom abstraction, the incipient substrate radical and rebound intermediate comprise a caged radical pair [Fe–OH **·**R]. We note that weak interactions between the substrate radical and the iron center at this stage would facilitate spin state interconversions for the ensemble. Another important feature of such caged radical species is the competition between the in-cage radical recombination and the diffusive cage escape. In an enzyme active site a water molecule, or a protein functional group, can insert itself between the substrate radical and the metal center as in [Fe–OH···OH_2_·R]. There is abundant photophysical evidence for competitive cage-escape and recombination as stochastic events in radical reactions since their initial discovery in 1930s [123]. A compelling example is the homolytic cleavage of carbon-cobalt bond in 5′-deoxyadenosylcobalamin (coenzyme B_12_) to form cobalt(II)-cobalamin and an adenosyl radical, which is important for the biological functions of coenzyme B_12_-dependent mutase enzymes. Time-resolved spectroscopic studies showed that the initial radical pair formed after Co–C bond homolysis in adenosylcobalamin undergoes in-cage radical recombination and cage escape both at approximately 10^9^ s^−1^, clearly indicating a competition between the two processes [124–126].

The realization of the significant influence of cage effects in radical rebound processes came from our studies of synthetic heme-model compounds. In 1979, we reported the first synthetic iron porphyrin system that effected stereospecific alkane hydroxylation and olefin epoxidation [127]. Further examination of this reaction showed that in the presence of a radical trap, bromotrichloromethane, an 18% yield of the *bromination* trapping product was obtained [56]. This result clearly indicated the presence of the cage escaped substrate radical that had encountered BrCCl_3_ in solution.

**Fig. 11:**
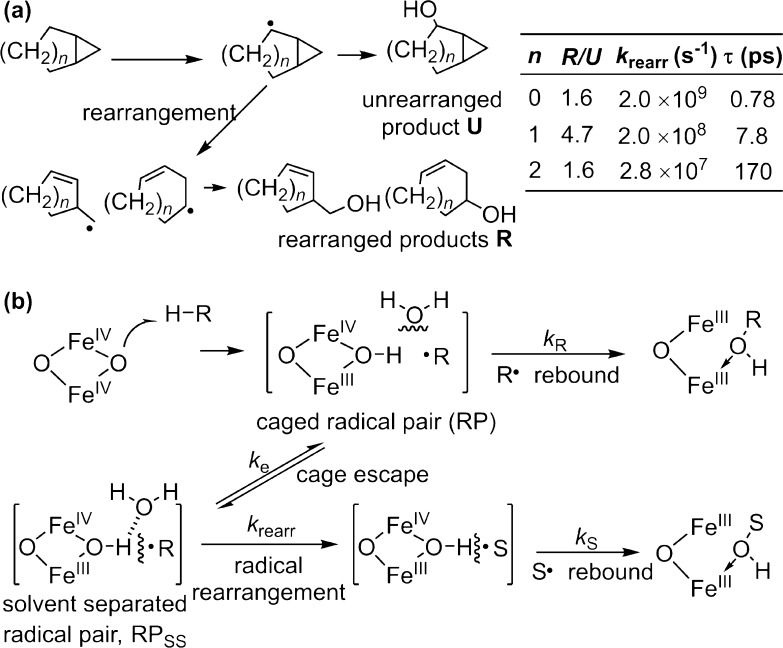
**a** Oxidation of radical clock probes with different rearrangement rate constants by non-heme diiron hydroxylase AlkB. **b** Schematic illustration of geminate recombination and cage escape. The scheme is adapted from Austin et al. [107]

The competition between cage escape and in cage recombination would offer an explanation to an enigma observed during the radical clock analysis of alkB, in which radical clock substrates with different rearrangement rate constants gave substantially different radical lifetimes (Fig. 11a) [107]. As shown in Fig. 11, three radical clocks, bicyclopentane, norcaranane, and bicyclo[3.1.0]hex-2-ane showed similar ratios of rearranged to unrearranged products (*R*/*U*) (1.6, 1.6, and 4.7), corresponding to apparent radical lifetimes of 0.78, 7.8, and 170 ns. These seemingly contradictory and counterintuitive results can be accommodated by a mechanism involving the diffusive cage escape of the substrate radical within the rather long enzyme active site to a solvent-separated radical pair, RP_ss_ (Fig. 11b) [108]. If the cage escape of the substrate radical occurs at a rate constant (*k*_e_) comparable to the rebound rate (*k*_R_), then for clocks with radical rearrangement constants (*k*_r_) slower than *k*_e_, the *R*/*U* ratios would not be much affected by the differences in *k*_r_ but reflect the “caging efficiency” *k*_e_/*k*_r_. This effect is analogous to the commonly observed suppression of the kinetic hydrogen isotope effects by strong binding of the substrate. The value of *k*_e_ is not expected to be constant because it will be substantially affected by the environment, such as viscosity of the solvent, tightness of the radical cage, steric and electronic environment within the active site and associative interactions of the substrate with the enzyme. An example is the large effect of substrate concentration on apparent 2-norcaranyl radical lifetime for AlkB. In this case, the presence of additional substrate molecules could displace and reorganize active-site water creating a bulkier hydrophobic environment that reduces *k*_e_ and facilitates the recombination of the initial radical pair. An important lesson learned here is that while diagnostic substrates can be used to detect the presence of intermediate radicals, the observed *R*/*U* ratios do not reflect the rate of rebound step in a simple way.

**Fig. 12:**
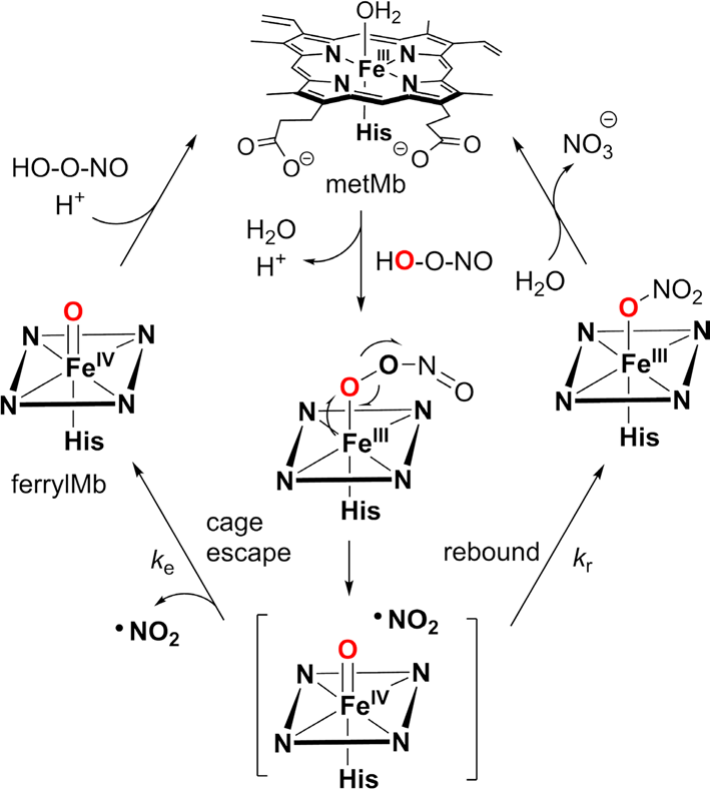
Catalytic cycle of metMb-catalyzed peroxynitrite decomposition

There are only a few studies on cage escape effects in P450-catalyzed hydroxylations [110]. But the degradation of peroxynitrite catalyzed by myoglobins provides an example to understand the cage escape/recombination processes in the context of the ferryl intermediates of hemeproteins (Fig. 12) [128, 129]. Myoglobins react rapidly with peroxynitrite to form a radical pair of a compound II ferryl intermediate and NO_2_, [MbFe^IV^=O** ·**NO_2_]. This radical pair can undergo recombination to form Fe^III^-heme and nitrate with rate constant *k*_r_, or NO_2_ can diffuse away from the distal heme site with rate constant *k*_e_. One manifestation of freely diffusing NO_2_ in this scenario is to induce tyrosine nitration elsewhere (i.e. in protein scaffold). This process is reminiscent of the recombination and escape events of deoxymyoglobin with O_2_, CO, and NO. The formation of MbFe^IV^=O was directly observed in these studies by stopped-flow spectrophotometry and a chemical trap, fluorescein, was used to capture the freely diffusing NO_2_. Based on these measurements, kinetic simulations revealed 10% cage escape of NO_2_ and *k*_e_/*k*_r_ = 0.10. Curiously, the reaction of oxymyoglobin with NO, which could have afforded the same peroxynitrito-iron(III) intermediate, did not afford detectable intermediates [130].

Very recently, Shaik and Nam have examined the cage escape/radical rebound processes for a number of non-heme metal-oxo systems [122]. They found that cage escape pathways generally showed low calculated energy barriers that could well compete with the in-cage radical recombination processes. In several cases, such as [Mn^IV^O(Bn-TPEN)]^2+^ and [Fe^IV^O(Bn-TPEN)]]^2+^ (Bn-TPEN = *N*-benzyl-*N*,*N*′,*N*′-tris(2-pyridylmethyl)-1,2-ethylenediamine), radical cage escape was found to be the preferred pathway and the diffusing radicals could be trapped by radical scavengers such as CCl_3_Br, *N*-bromosuccinamide, or O_2_ under aerobic conditions [131–134].

## Radical rebound mechanism: a reaction manifold for versatile biotransformations other than oxygenation

**Fig. 13:**
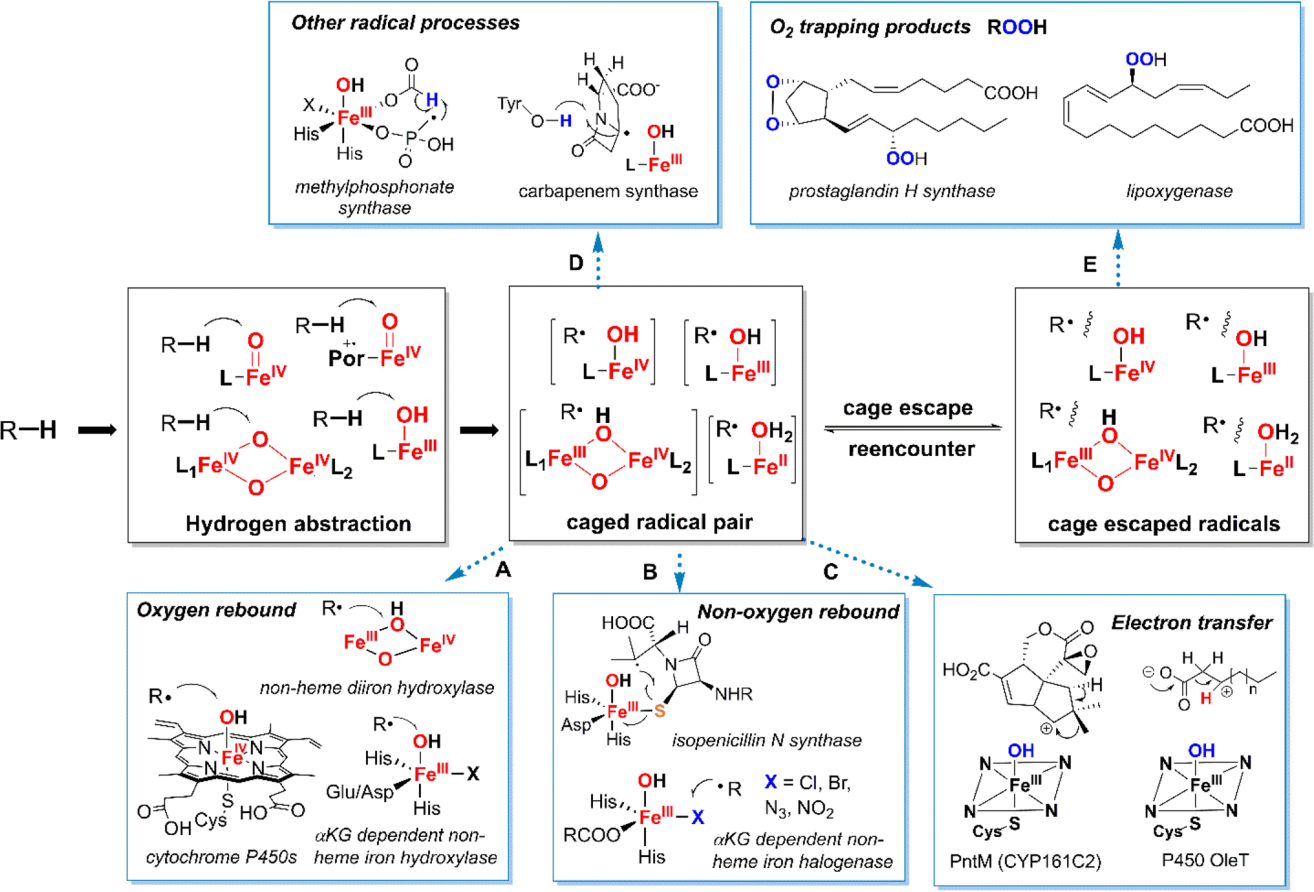
Generation of the radical pair via hydrogen atom abstraction and the various reaction outcomes depending on the behavior of the radical pair

The stepwise events of hydrogen abstraction and radical recombination in the rebound mechanism represents a general strategy exploited by nature to perform controllable radical reactions. As discussed in the previous section, the behavior of the incipient substrate radical is highly tunable. As such, the initial substrate radical can be employed in a variety of radical processes and lead to different reaction outcomes. In this context, oxygen rebound of substrate radicals to form a C–OH bond represents just one type of possible reactions enabled by the radical rebound mechanism. Indeed, nature has found ways to manipulate the behavior of the incipient radicals for chemistries other than oxygen rebound. Early examples include prostaglandin H synthase and lipoxygenase, in which incipient substrate radicals are trapped by oxygen to afford the hydroperoxy products (Fig. 13, pathway E). In these cases there are discrete oxygen-binding pockets within the protein that control the substrate radical rebound to a nearby oxygen molecule.

**Fig. 14:**
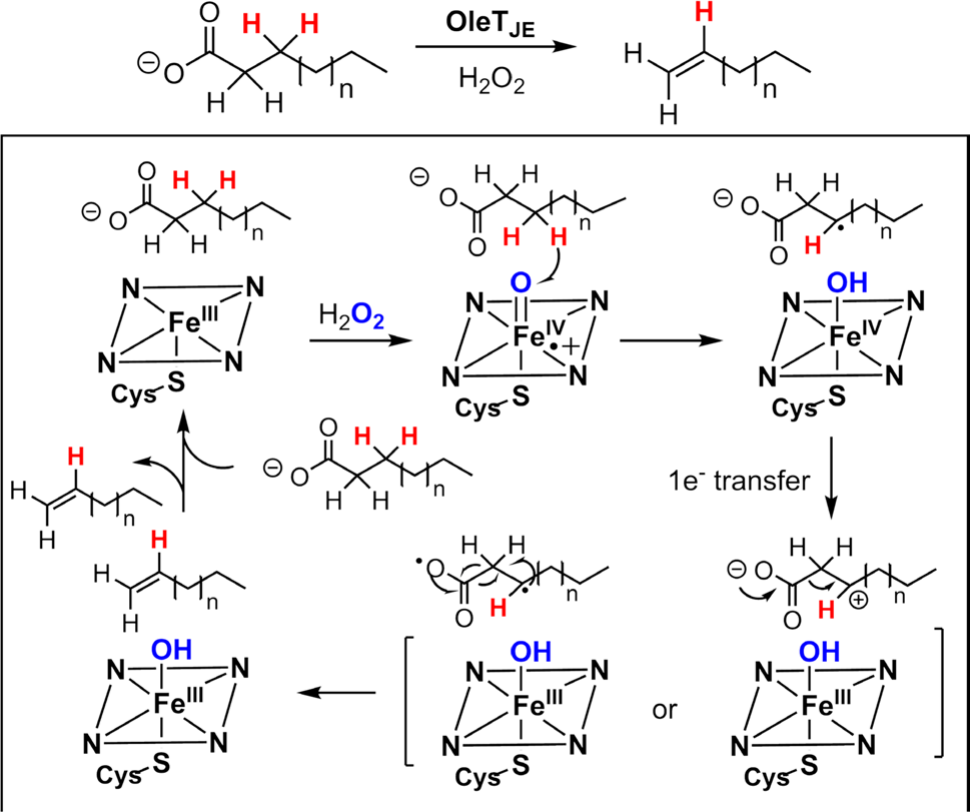
Oxidative decarboxylation catalyzed by OleT_JE_

In addition to these two enzymes, there are numerous examples described during the past decade highlighting the versatile reactions that incipient substrate radicals could participate. For example, diversion of the rebound intermediate to form olefins can be seen as a hydrogen atom rebound to the Fe^IV^–OH [32]. Another example is the conversion of fatty acids to terminal alkenes catalyzed by cytochrome P450 OleT_JE_ (Fig. 14). Since its discovery in 2011, this biotransformation has garnered considerable attention due to its potential applications in biofuel and fine chemical synthesis [135–137]. The studies of Makris et al. showed that the mechanism of OleT_JE_-catalyzed decarboxylation involved an initial hydrogen abstraction by OleT_JE_ compound I as in P450-catalyzed hydroxylations at the β carbon relative to the carboxyl group [138, 139]. This result is intriguing, as it suggests that the usual oxygen rebound step must be inhibited in the case of OleT and the incipient radical is redirected to a C–C bond scission pathway, presumably involving single electron transfer from substrate radical to compound II and subsequent loss of CO_2_ to form C=C bond. Such electron transfer processes to form substrate cation intermediate have also been reported recently for CYP161C2 (PntM), which catalyzes an oxidative rearrangement reaction that converts pentalenolactone F to pentalenolactone (pathway C, Fig. 13) [140].

Computational studies by de Visser et al. also suggested a slow-down of the radical rebound for OleT [141]. An 8 kcal/mol energy barrier was estimated for the oxygen rebound of substrate radical to OleT_JE_ compound II, while the energy barrier of the decarboxylation pathway was estimated to be below 1 kcal/mol. The calculation also suggested that hydrogen bonding interactions within the P450 OleT_JE_ active site are essential for the destabilization of the oxygen rebound.

**Fig. 15:**
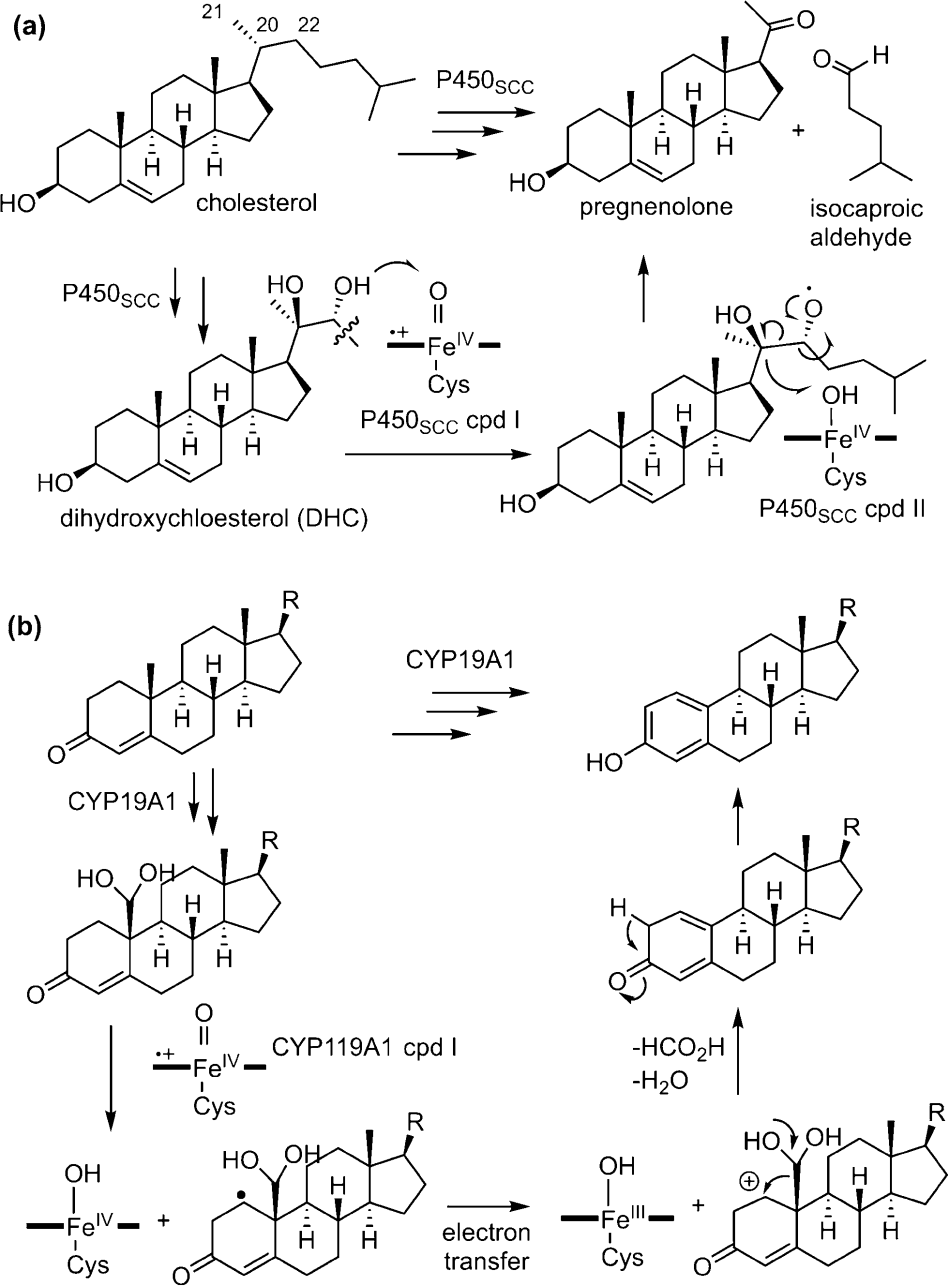
**a** Mechanism of the C–C scission of DHC catalyzed by P450_SCC_. **b** Proposed mechanism of the aromatization step in the conversion of androgens to estrogens catalyzed by CYP19A1

An analogous example to P450 OleT_JE_-catalyzed decarboxylation is the iconic side chain C–C bond scission reaction catalyzed by cytochrome P450_SCC_, which converts cholesterol to pregnenolone. P450_SCC_ first performs two successive hydroxylations of cholesterol to form 20α,22(*R*)-dihydroxycholesterol (DHC) (Fig. 15a). In the third step, P450_SCC_ compound I abstracts the hydrogen from the C22 hydroxyl group to form a C22-oxy radical and compound II. The C22-oxy radical then undergoes C20–C22 bond homolysis to afford isocaproaldehyde and a C20 ketyl radical that recombines with compound II to afford pregnenolone [142]. Recent reevaluation of the activity of steroid aromatase CYP19A1 also suggests a mechanism involving the redirection of the oxygen rebound (Fig. 15b) [143]. In this mechanism, the radical intermediate generated after the initial hydrogen abstraction undergoes one-electron transfer to form a cation in the A ring of androgen, which subsequently aromatizes to form the estrogen. From these examples, it is clear that the radical pair [Fe–OH** ·**R] generated after the initial C–H bond cleavage follows different reaction channels leading to novel reactivities other than oxygenation.

**Fig. 16:**
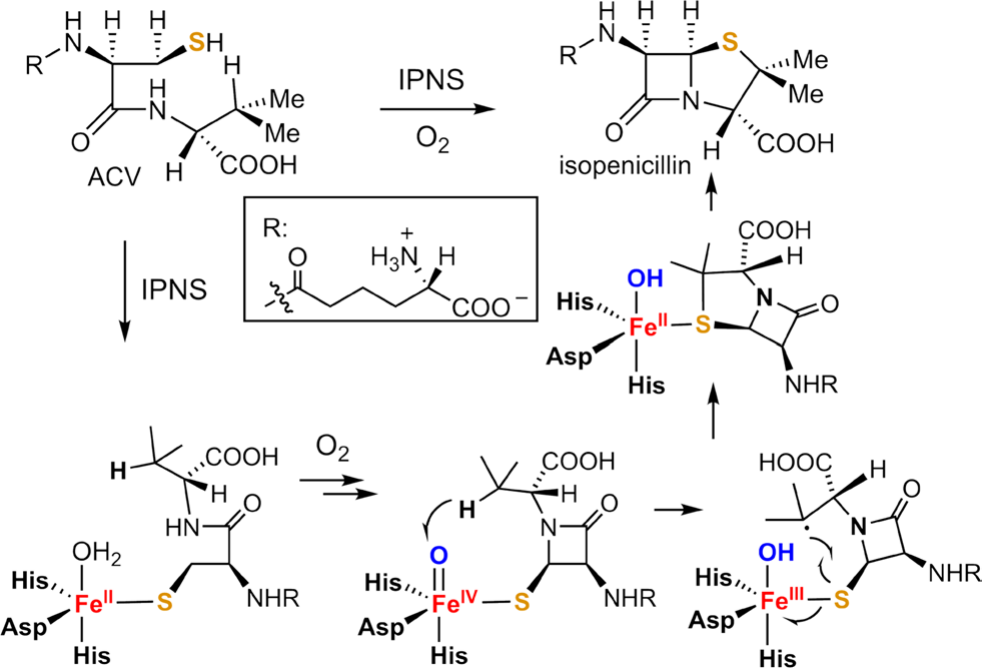
Mechanism of IPNS-catalyzed formation of isopenicillin from ACV

Steering radical rebound is also widely exploited in non-heme iron enzymes. A compelling example is the formation of isopenicillin N from δ-(l-α-aminoadipoyl)-l-cysteinyl-d-valine (ACV) catalyzed by isopenicillin N synthase (IPNS) (Fig. 16), which is a key step in the biosynthesis of penicillin and cephalosporin antibiotics [144]. IPNS is a non-heme iron(II)-dependent oxygenase with an active site containing an iron coordinated to a 2-His-1-carboxylate facial triad. The elegant work of Baldwin and co-workers showed that two stages were involved for the formation of isopenicillin N from ACV [144]. The first stage involves the formation of the β-lactam ring and an Fe^IV^=O intermediate. In the second stage, the Fe^IV^=O intermediate abstracts a hydrogen from the valinyl C3–H and the resulting C3 carbon radical recombines with the iron-bound sulfur to generate the thiazolidine ring, a process analogous to the oxygen rebound in oxygenation reactions [145]. The involvement of the radical intermediate in this process was illuminated by employing a cyclopropane-containing ACV analog and observation of a rearranged product of the cyclopropylcarbinyl radical [146]. The result that IPNS selectively forms a C–S bond rather than C–O bond during the radical rebound step is intriguing, as the C–S bond is much weaker than the C–O bond. The DFT studies by Morokuma et al. on IPNS active-site models showed that, although the formation of hydroxylation product is more thermodynamically favorable, the C–S bond formation is kinetically favored as the transition state for sulfur rebound is 3.7 kcal/mol lower than oxygen rebound barrier [147–149].

**Fig. 17:**
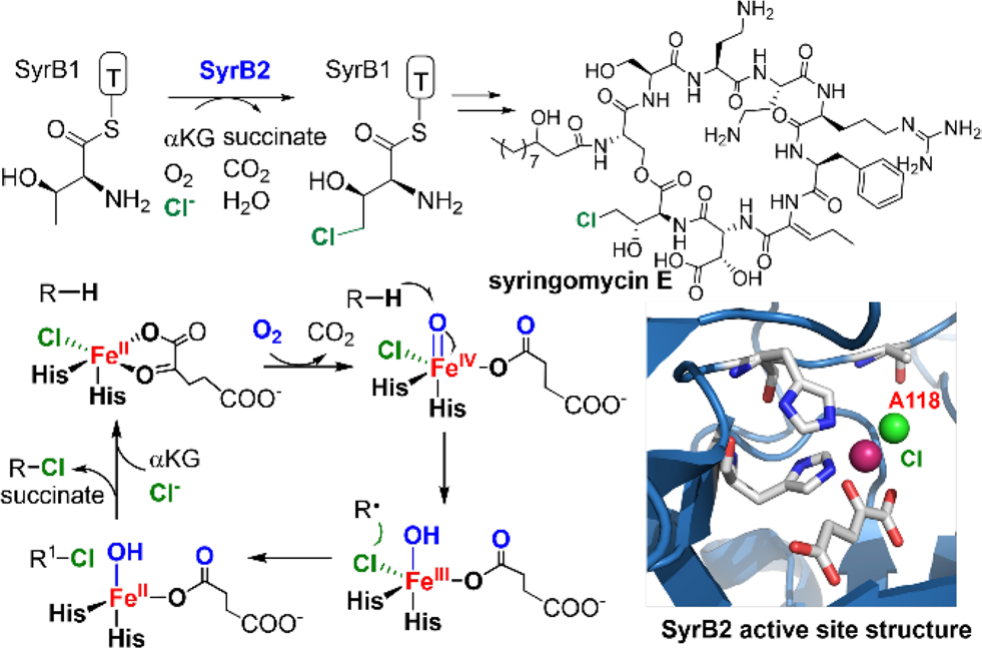
Mechanism of C–H chlorination catalyzed by SyrB2 (PDB: 2FCT). Colors: iron (*magenta*), oxygen (*red*), nitrogen (*blue*), chlorine (*green*)

Aliphatic C–H chlorination reactions catalyzed by *α*-ketoglutarate (*α*KG) dependent non-heme iron(II) halogenases represent another example of non-oxygen atom rebound. The first Fe(II)/*α*KG halogenase discovered is the halogenase SyrB2, which catalyzes the C4 chlorination of threonine appended to the carrier protein SyrB1 (l-Thr-SyrB1) during the biosynthesis of syringomycin E by *Pseudomonas syringae* (Fig. 17) [150]. Structural studies showed that, in Fe(II)/*α*KG halogenases, the ferrous center is coordinated to two histidines and a chloride instead of the 2-His-1-carboxylate coordination in a typical Fe(II)/*α*KG hydroxylase [151]. A conserved alanine or glycine was found at the position of aspartate or glutamate in Fe(II)/*α*KG hydroxylase which created the space for chloride binding. The mechanism of Fe(II)/*α*KG halogenases involves a chloroferryl intermediate (Cl–Fe^IV^=O) that abstracts a hydrogen atom to form a substrate radical and a chlorohydroxoferric intermediate (Cl–Fe^III^–OH). The substrate radical then rebounds to the chlorine to afford chlorination product.

**Fig. 18:**
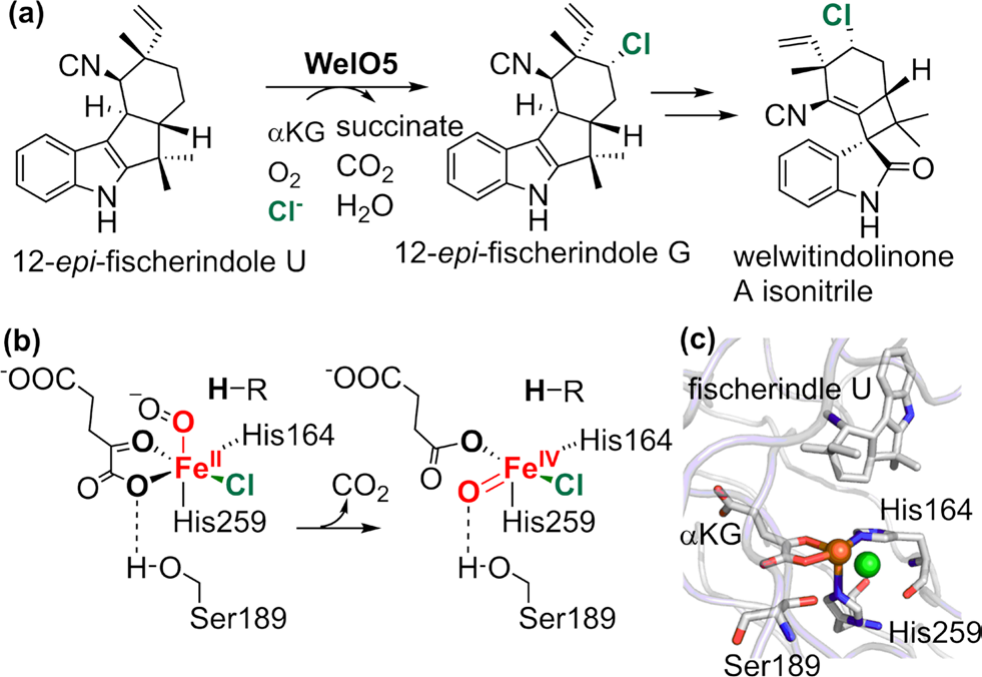
**a** C–H Chlorination of 12-epi-fischerindole U catalyzed by the halogenase WelO5. **b** Relocation of the iron-oxo unit in the formation of oxoiron(IV) intermediate. **c** Active site structure of WelO5 (PDB: 5IQT). Colors: iron (*orange*), oxygen (*red*), nitrogen (*blue*), chlorine (*green*)

The factors leading to the preference for chlorine over hydroxyl transfer during the rebound scenario has attracted considerable attention. A number of studies involving spectroscopic and structural analysis as well as reactivity assays using native substrate analogs show that a closer positioning of the substrate radical to the chlorine during the rebound is an important factor in determining the chlorination selectivity [152–158]. Very recently, Boal and Liu obtained the first substrate-bound crystal structure of a carrier protein-independent Fe(II)/*α*KG halogenase, WelO5, which chlorinates 12-*epi*-fischerindole U at the C13 position [159–161]. The crystal structure indicated a relocation of the oxo unit from the axial position to the equatorial plane defined by the chloride and the *α*KG, which would draw the oxo group further way from the substrate (Fig. 18). This transition is apparently aided by a hydrogen-bonding interaction with the hydroxyl group of Ser189 in the Fe(II) secondary sphere. Mutation of Ser189 to alanine led to an equal portion of hydroxylation and chlorination products.

Chlorination is not the only reaction that Fe(II)/*α*KG halogenase could effect. Recently, Bollinger and co-workers found that Fe(II)/*α*KG halogenases could catalyze C–H azidation and nitration upon replacing the iron-bound chloride with azide and nitrite [162]. Analogous to the chlorine rebound scenario in halogenases, the incipient radical formed after initial C–H abstraction by Fe^IV^=O recombined with an azidoferric (N_3_–Fe^III^–OH) or a nitritoferric (NO_2_–Fe^III^–OH) intermediate to form the C–N_3_ or C–NO_2_ bond, albeit in modest conversions.

**Fig. 19:**
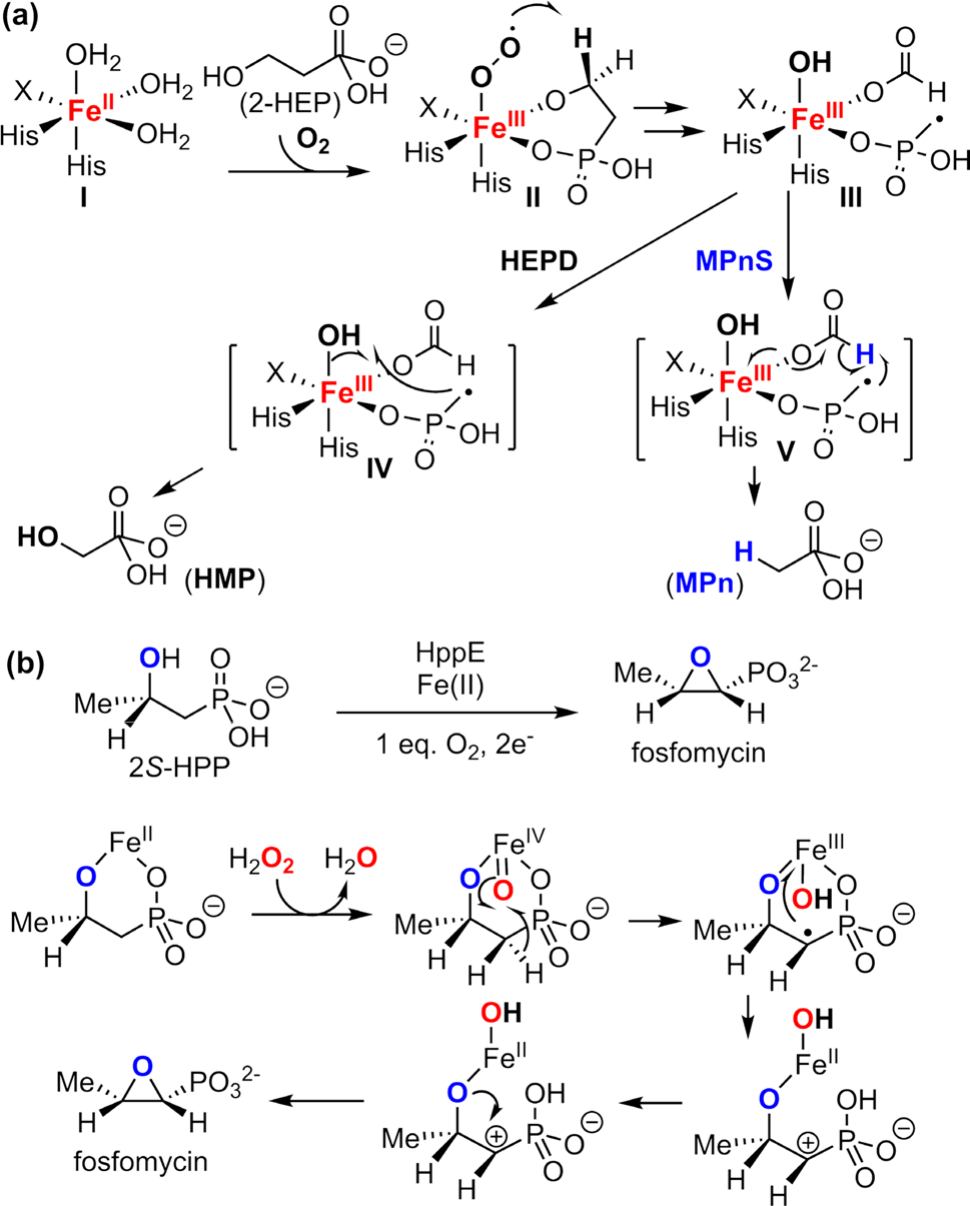
**a** Synthesis of HMP and MPn from 2-HEP catalyzed by HEPD and MPnS respectively. **b** Synthesis of fosfomycin from 2S-HPP catalyzed by HppE

Non-heme iron oxygenases can also catalyze other non-oxygenation reactions by employing substrate radicals in different radical processes. An interesting example is the transformation of 2-hydroxyethylphosphonate (2-HEP) to different products by enzymes sharing a similar mechanism [163–165]. As shown in Fig. 19, 2-HEP can be converted to methylphosphonate (MPn) by MPn synthase (MPnS) or to hydroxymethylphosphonate (HMP) by 2-HEP dioxygenase (HEPD). These two reactions proceed through a common radical intermediate (species **III** in Fig. 19a). In HEPD, HMP was obtained by the oxygen rebound of the methylphosphonate radical to the Fe^III^–OH complex (**IV** in Fig. 19a), whereas in MPnS, the methylphosphonate radical will abstract a hydrogen from the formate to afford MPn (**V** in Fig. 19a).

An analogous example of HEPD and MPnS is 2-hydroxypropylphosphonate epoxidase (HppE) [166–170]. HppE is involved in the biosynthesis of the antibiotic fosfomycin from (*S*)-2-hydroxypropylphosphonate (2*S*-HPP). In this reaction, an oxoferryl intermediate is formed prior to the epoxide formation step that abstracts the *pro*-*R* hydrogen from C1 of 2*S*-HPP. Subsequent electron transfer from the C1 radical to Fe^III^–OH and epoxide ring closure afford the fosfomycin product (Fig. 19b).

In addition to the examples discussed herein, there are a number of other reactions that can be effected by iron oxygenases via controlling the reaction pathways of incipient radicals, including stereoinversion, oxacyclization, carbodesaturation, etc. In another variation of the rebound spectrum of reactions, aldehyde deformylation by the diiron enzyme ADO produces alkanes in a process that can be seen as a hydrogen rebound from Fe–OH_2_ [171]. These reactions have recently been reviewed by Bollinger and Krebs [172]. The common feature of these biotransformations is the involvement of an initial hydrogen atom abstraction by high-valent iron-oxo intermediates. The desired reaction outcome is achieved by directing the incipient substrate radicals to the corresponding radical reaction pathways such as oxygen rebound, non-oxygen atom rebound, electron transfer, radical cage escape, etc. In this way, nature can achieve a diverse array of C–H transformations with molecular oxygen as the oxidant by employing these aspects of the rebound process.

## Harnessing the radical rebound mechanism for novel organic radical transformations through biomimetic catalysis

The direct functionalization of aliphatic C–H bonds remains one of the grand challenges for the chemical catalysis community [173]. Activation of aliphatic C–H bonds with organometallic reagents, such as Shilov chemistry or many Pd-catalyzed C–H functionalizations, generally requires harsh conditions or utilizes a directing group approach because of the low acidity and weak coordination capability of aliphatic C–H bonds [174–177]. On the other hand, it has been long recognized that direct functionalization of aliphatic C–H bonds can be achieved via radical chemistry [178–180]. A classic example is the radical chlorination of hydrocarbons with Cl_2_ under photolytic or thermal conditions, which have been used for the industrial synthesis of chloroform and dichloromethane from methane [181]. The power of radical C–H activation has been further demonstrated by the recent renaissance of catalytic radical-based methods, especially photoredox catalysis that has provided a variety of new methods for aliphatic C–H functionalization reactions [182–184].

**Fig. 20:**
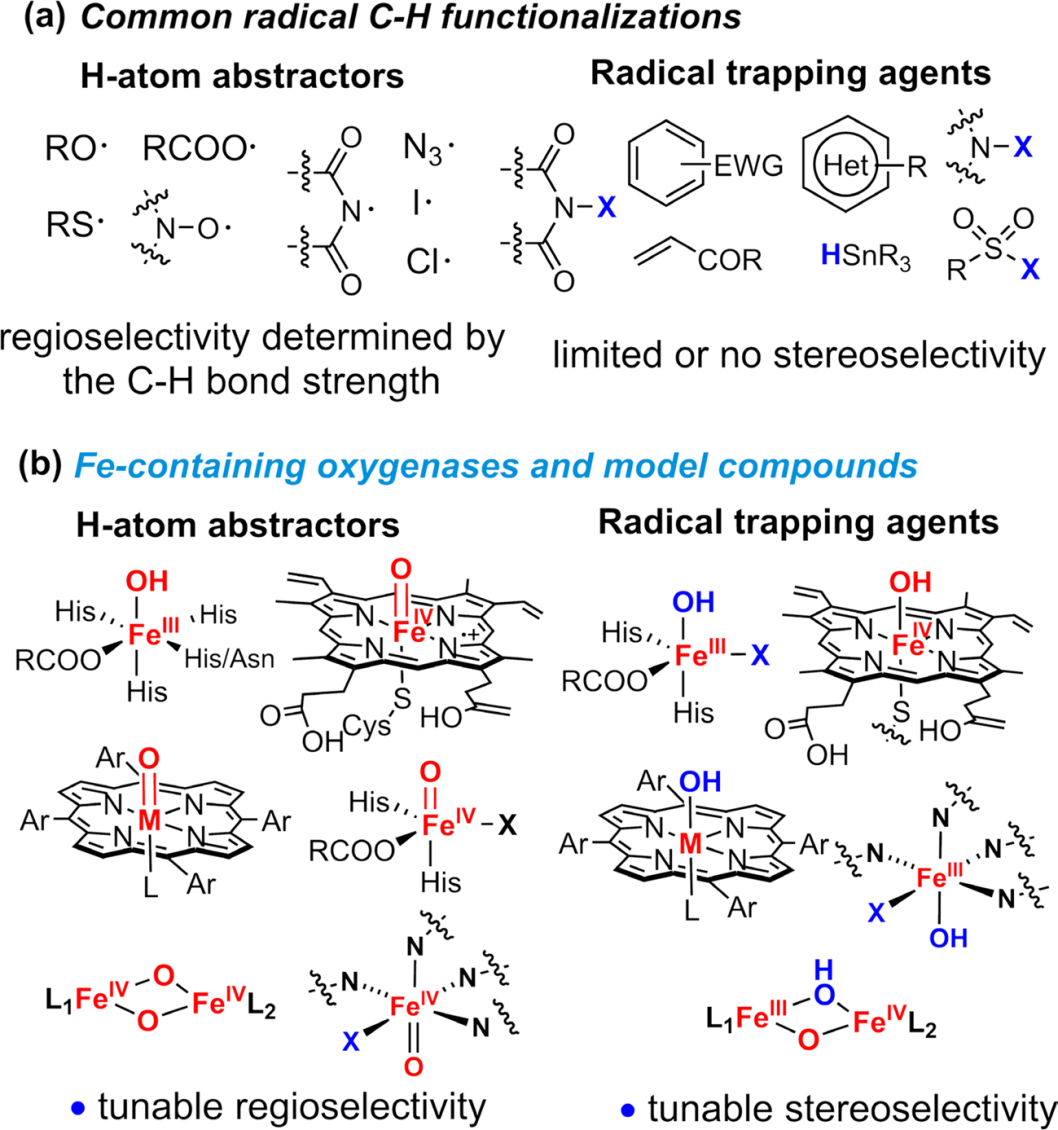
Comparison between common radical C–H functionalizations (**a**) and those mediated by iron oxygenases and their model compounds (**b**)

In this context, the radical rebound strategy employed by iron-containing oxygenases represents a novel and general paradigm for effecting direct aliphatic C–H functionalizations. There are three main features that set this biological approach apart from other radical C–H activation methodologies (Fig. 20). First, the C–H activation is mediated by first-row metal complexes such as metal-oxo intermediates instead of organic or inorganic radicals commonly seen in radical reactions. The use of metal-based hydrogen abstracting intermediates enables the control of regioselectivity of C–H activation beyond the innate reactivity of C–H bonds. With this approach, stronger primary or secondary C–H bonds can be selectively functionalized in the presence of much weaker tertiary C–H bonds. Second, in a radical rebound approach, the new bond formation step of carbon-centered radical also proceeds through a metal-based intermediate, allowing for highly stereoselective trapping of the carbon-centered radicals. This feature resembles several photoredox reactions in which a nickel or copper catalyst can trap the alkyl radicals and effect enantioselective C–C or C–N bond formations. Another feature of the radical rebound approach is the controlled generation of carbon-centered radicals rather than radical chain mechanisms for many organic radical transformations. In biology, this feature is critical as freely diffusing carbon radicals are harmful to the biological system. This characteristic is also desired for organic transformations as it will eliminate unwanted side reactions.

There are many catalytic systems for aliphatic C–H hydroxylation reactions based on synthetic models of Fe-containing oxygenases, which have been reviewed extensively [17, 18, 185]. Herein, we will mainly focus on nonoxygenation reactions via the radical rebound mechanism, which has offered solutions to several most challenging reactions in synthetic organic chemistry.

**Fig. 21:**
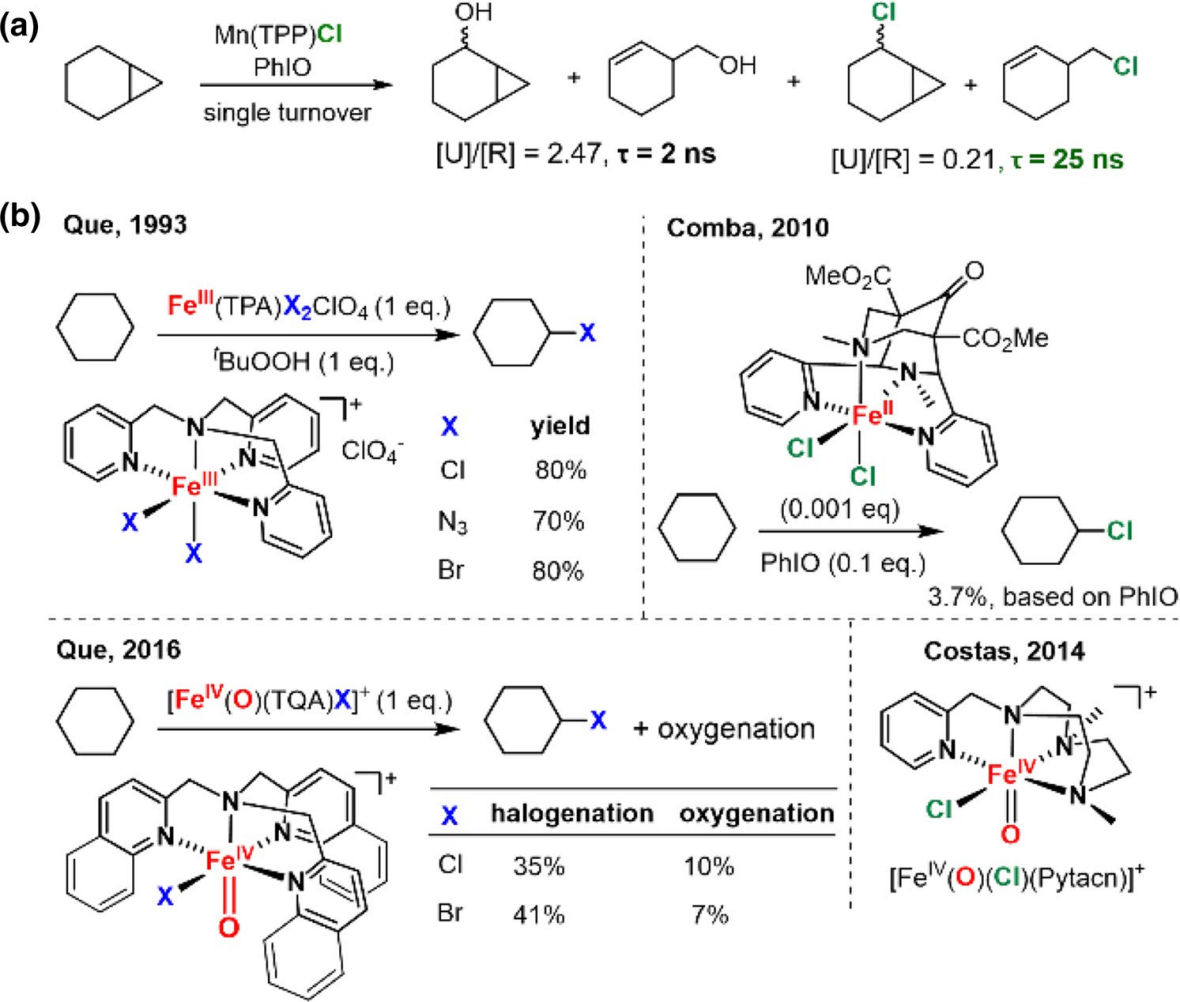
**a** Detection of the chlorination product in alkane hydroxylation catalyzed by a manganese porphyrin. **b** Halogenation reactivity of several non-heme iron model compounds

The first hint of such nonoxygenation reactions was obtained during our investigation of synthetic models for P450s. In 1980, we found that manganese porphyrins can effect highly efficient alkane hydroxylations. In this reaction, side chlorination products were also observed, which were determined to derive from the chloride axial ligand on the manganese porphyrin catalyst [55]. The mechanistically diagnostic radical clock norcarane used in that study clearly showed the presence of the radical rearranged chlorination product, 3-chloromethyl-cyclohexene. This chlorination product was formed through a hydrogen abstraction/radical rebound pathway with a radical lifetime of ~25 ns (Fig. 21a). Subsequent studies by our group and the Hill group showed that a variety of axial ligands, including bromide, iodide, and azide, could undergo this oxidative ligand transfer reaction [186–188]. Meunier and coworkers also found that manganese porphyrin/NaOCl system could effect C–H chlorination of cyclohexane [189]. In 1993, Que and co-workers demonstrated that such ligand transfer reaction also occurred with non-heme iron complexes [190]. Very recently, several oxoiron(IV) non-heme complexes with a cis-coordinated chloride ligand have been examined because of their resemblance to the halogenation intermediate of iron halogenases (Fig. 21b) [191–193]. Reactivity studies revealed that some of these complexes can effect aliphatic C–H chlorination with a ratio of oxygenation to halogenation varying from ~1 to ~6.

**Fig. 22:**
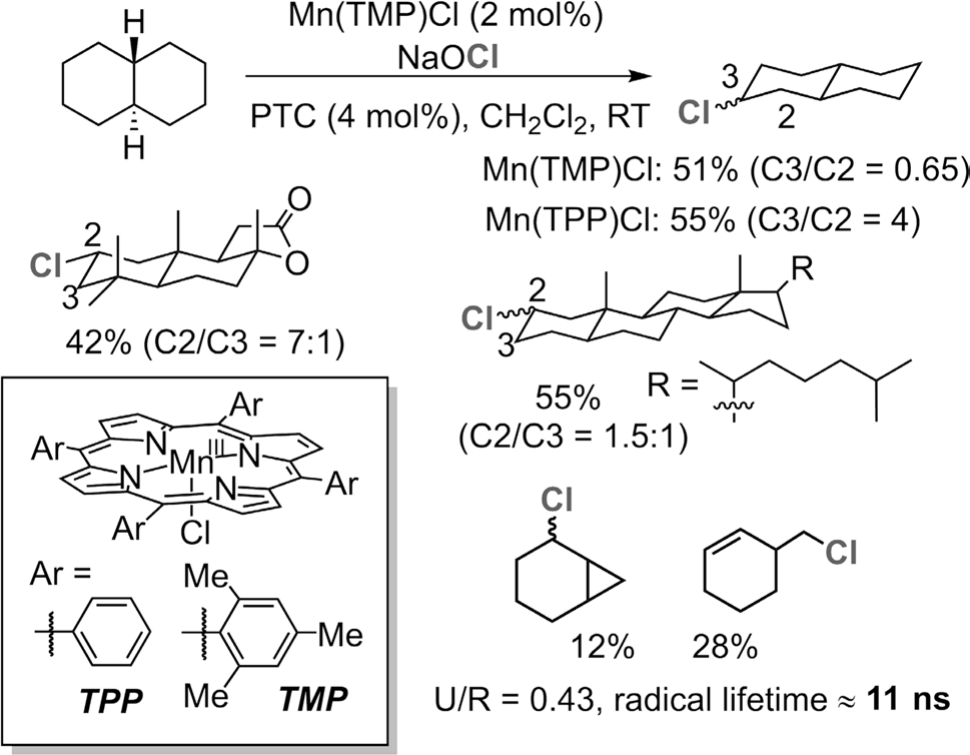
Aliphatic C–H chlorination catalyzed by manganese porphyrins

A standing challenge for implementing the radical rebound approach for catalytic C–H functionalization has been the suppression of the oxygenation products formed via oxygen rebound. The first breakthrough was achieved in 2010, when we discovered an unusual manganese porphyrin-catalyzed aliphatic C–H chlorination reaction (Fig. 22) [194]. With sodium hypochlorite as the oxidant and chlorine source, a variety of hydrocarbons were readily chlorinated with only trace amount of oxygenation products. Product selectivities were very much different than those of typical chlorination reactions. The chlorination of *trans*-decalin, for example, afforded mainly secondary chlorination products (2°/3° > 9), which is in drastic contrast to the chlorination of the same substrate with *N*-chlorosuccinimide (NCS) or hypochlorous acid (2°/3° ratio ~0.7 and 0.3). Furthermore, the sterically bulky Mn(TMP)Cl catalyst yielded chlorination products with a C3/C2 ratio of 4, much higher than that of the less sterically hindered Mn(TPP)Cl (C3/C2 = 0.65). These results demonstrated that the regioselectivity of these radical C–H chlorination reactions can be regulated by the steric and electronic properties of the catalyst, hints of which were seen in the very early hydroxylation work (Fig. 6) [55, 56]. This catalytic chlorination also showed high stereoselectivities during the chlorine transfer step. For example, 5α-cholestane was selectively chlorinated at C2 and C3 positions with equatorial to axial ratio of 15:1 and 1:2, respectively.

Radical clock studies showed that the reaction followed a radical mechanism with a radical lifetime ~11 ns, which is much longer than that of the analogous hydroxylation (~2 ns). No cation rearranged product was observed, which further indicated a radical rebound mechanism.

**Fig. 23:**
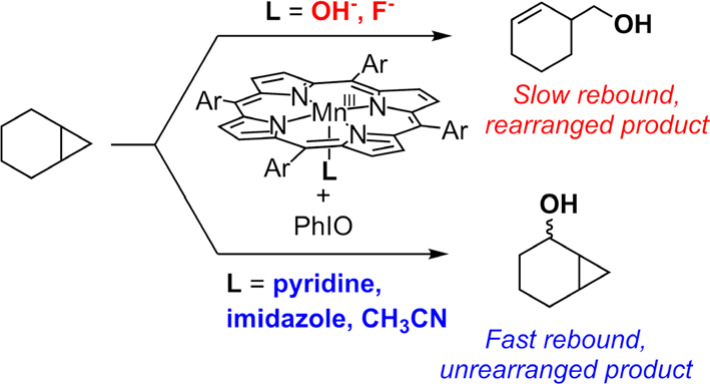
Effect of axial ligands on the oxygen rebound step

An intriguing question is what slows down the oxygen rebound and inhibits the oxygenation products in this chlorination reaction. Later studies showed that the axial ligand played an important role in determining the radical lifetime of the incipient carbon radicals (Fig. 23). The addition of strongly ligating imidazole or pyridine to the chlorination system significantly suppressed chlorination and led to oxygenation products instead [195]. This axial ligand effect was further supported by the radical clock analysis of the Mn-catalyzed hydroxylation reaction in which radical lifetime increased significantly by changing the axial ligand from imidazole or pyridine to anionic σ-donating ligands hydroxide and fluoride.

**Fig. 24:**
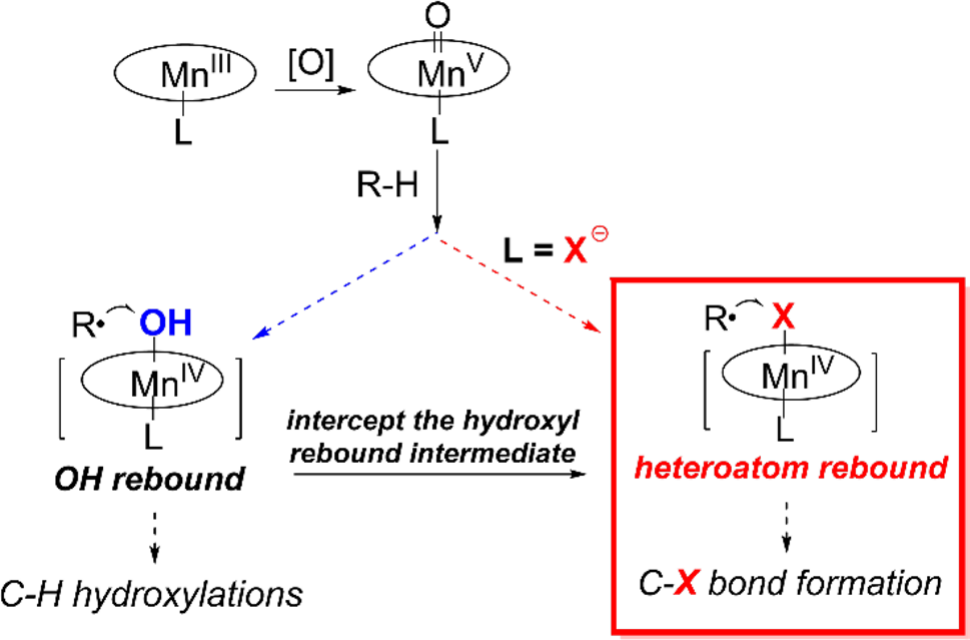
The concept of heteroatom rebound catalysis (HRC) for developing C–H functionalization reactions

**Fig. 25:**
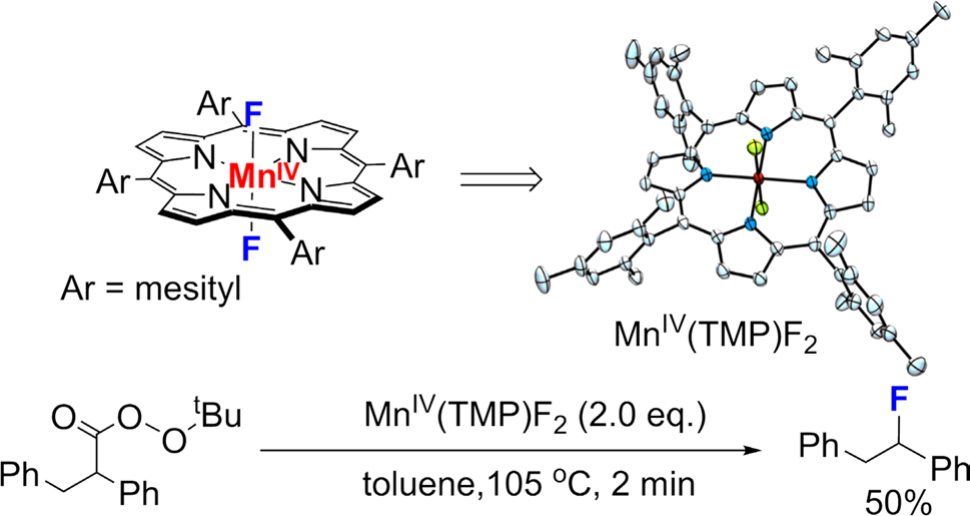
Trapping of alkyl radicals by *trans*-Mn^IV^(TMP)F_2_ via fluorine atom transfer. X-ray crystal structure of *trans*-Mn^IV^(TMP)F_2_ drawn at 50% probability of the electron density with following atom colors: F (*yellow*), Mn (*magenta*), N (*blue*), C (*cyan*) (H atoms are omitted for clarity)

This unprecedented axial ligand effect on radical rebound offered a general strategy to develop biomimetic radical C–H functionalization reactions that nature had not yet devised via the control of the radical rebound step (Fig. 24). A good demonstration of this approach is the development of an unprecedented aliphatic C–H fluorination with fluoride ion developed by our group in 2012 [196]. The rationale of this reaction is that the addition of fluoride ion would suppress oxygen rebound and the incipient radical could be redirected to recombine with a Mn^IV^–F intermediate in a process that resembles oxygen rebound. Although there are many examples of radical fluorination based on electrophilic fluorination reagents [197–200], radical fluorination with fluoride-derived Mn–F rebound was unprecedented. A significant challenge to developing fluoride-based radical fluorination lies in the high oxidation potential of fluoride ion that typically prevents it from participating in redox chemistry [201–203]. We found that binding of fluoride ion to a manganese(IV) porphyrin activated it toward fluorine atom transfer to the substrate alkyl radical. To probe this idea, a unique *trans*-difluoromanganese(IV) porphyrin complex was synthesized and isolated. Treating this species with radical precursors generated under thermolytic conditions afforded alkyl fluoride products via radical trapping in high yields (Fig. 25).

**Fig. 26:**
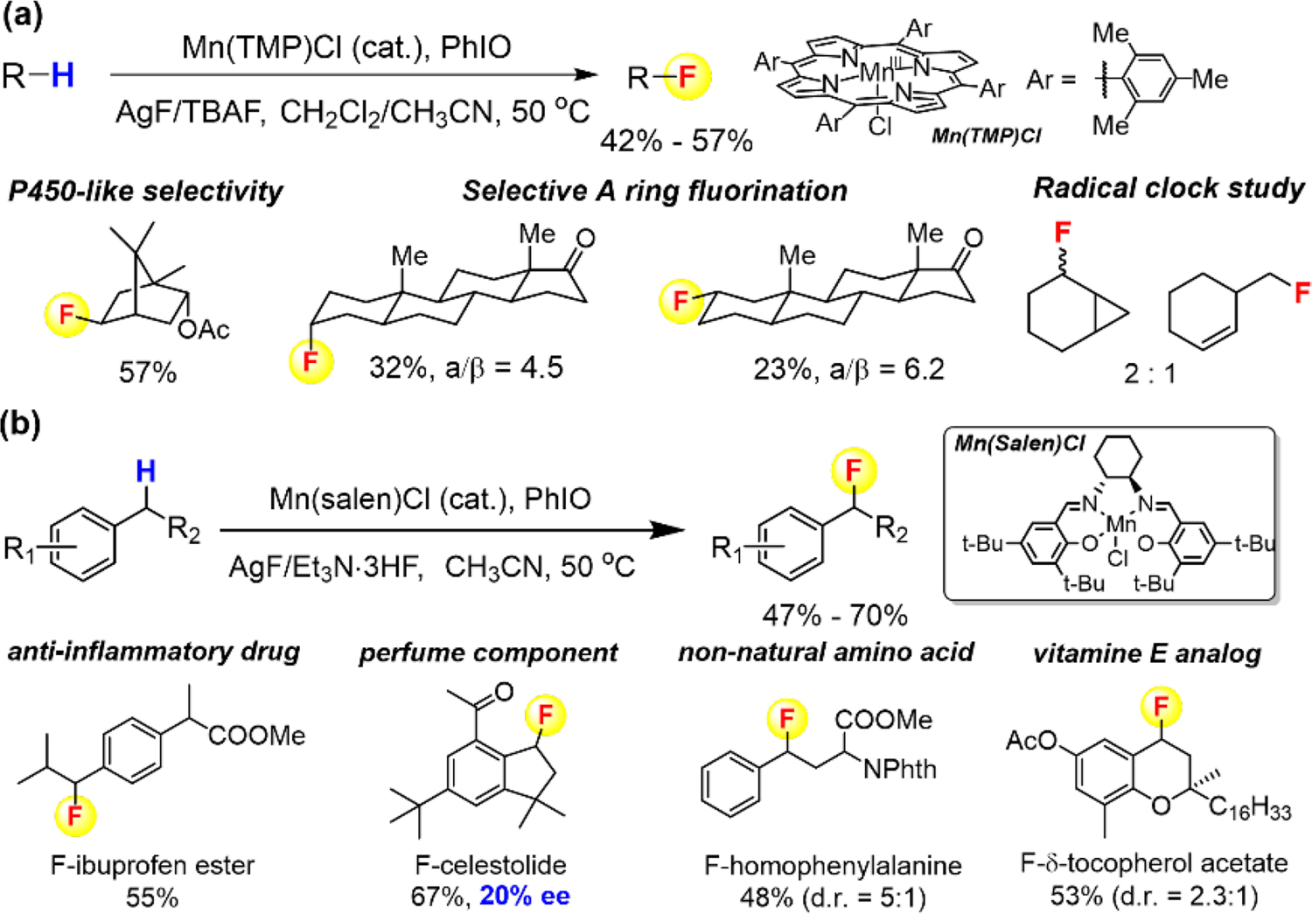
**a** Aliphatic C–H fluorination catalyzed by manganese porphyrins. **b** Benzylic C–H fluorination catalyzed by magnanese salens

With this mechanistic insight in hand, we developed the first fluoride-based aliphatic C–H fluorination reaction (Fig. 26a) [196]. Like the manganese porphyrin-catalyzed chlorination, this reaction showed catalyst-controlled regioselectivity with a preference for secondary aliphatic C–H bonds in the presence of weaker tertiary C–H bonds. High regio- and stereo-selectivity were achieved for a number of complex substrates including the terpenoid sclareolide and a steroid, 5α-androstan-17-one. Radical clock studies again showed a radical rebound mechanism with radical lifetimes of ~2 ns. Highly selective benzylic C–H fluorination was achieved by changing the catalyst to manganese salens (Fig. 26b). When using a chiral manganese salen catalyst, a 20% ee was detected for the fluorination of celestolide. This small but mechanistically revealing enantioselectivity led strong support for the radical rebound to a catalyst-bound Mn–F intermediate. DFT calculations to probe this process predicted a very low energy barrier (~3 kcal/mol) for such a fluorine rebound process from manganese.

**Fig. 27:**
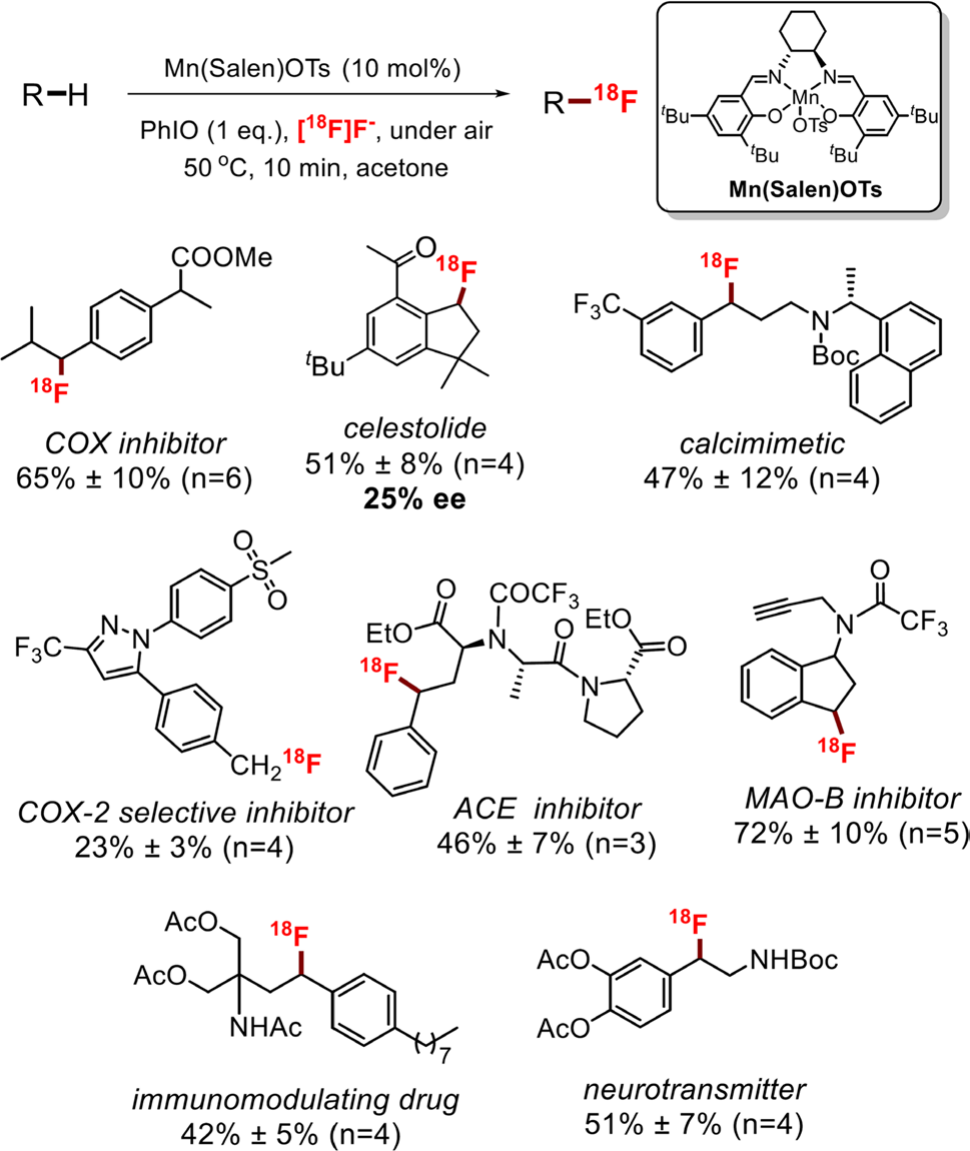
Aliphatic C–H ^18^F labeling mediated by manganese salen catalysts

The use of fluoride ion in this C–H fluorination reaction allowed us to develop the first ^18^F labeling reaction of aliphatic C–H bonds using [^18^F]fluoride, which could have wide applications in positron emission tomography (PET) (Fig. 27) [204]. [^18^F]fluoride is the preferred ^18^F source for molecular labeling with ^18^F for PET because of its high specific activity (a measure of ^18^F/^19^F ratio in the labeled molecules) [205]. The major challenge of this labeling reaction is the short half-life of the ^18^F radionuclide (~110 min) and its minuscule quantities (pmol–nmol) under typical radiochemical process conditions [206, 207]. We found that manganese salen catalysts with a labile tosylate ligand could efficiently trap pmol of ^18^F in the solution with over 90% efficiency. With this approach, our labeling method effects efficient C–H ^18^F labeling of a variety of substrates including nine complex drug molecules or drug analogs within 10 min with very practical radiochemical conversions (conversion of ^18^F into the target molecule) up to 70%. Mechanistic studies showed that this reaction also proceeded through an H-abstraction/fluorine rebound mechanism as a 25% ee was observed for the ^18^F labeling of celestolide with chiral manganese salen catalyst. The success of this ^18^F labeling reaction provided critical insight to the fluorine rebound step. It is worth noting that in ^18^F reactions, all other reactants, including the manganese ‘catalyst’, are in vast excess because of the tiny quantity of ^18^F fluoride (pmol). Therefore, the concentration of the Mn^IV^–^18^F intermediate would be much lower that of Mn^IV^–OH intermediate. However, the observed high ^18^F conversion into target molecule suggests that the radical rebound to Mn^IV^–^18^F must be highly selective in capturing incipient alkyl radicals.

**Fig. 28:**
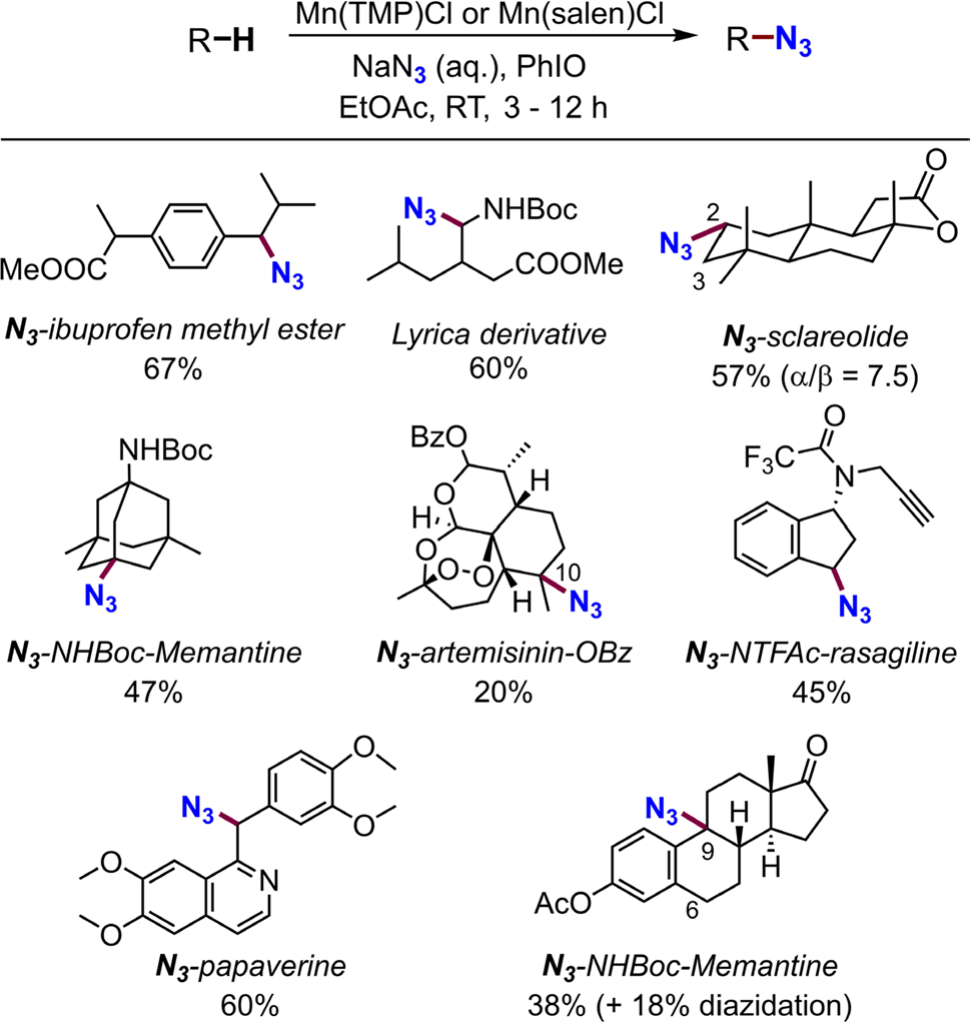
Manganese-catalyzed late-stage aliphatic C–H azidation

**Fig. 29:**
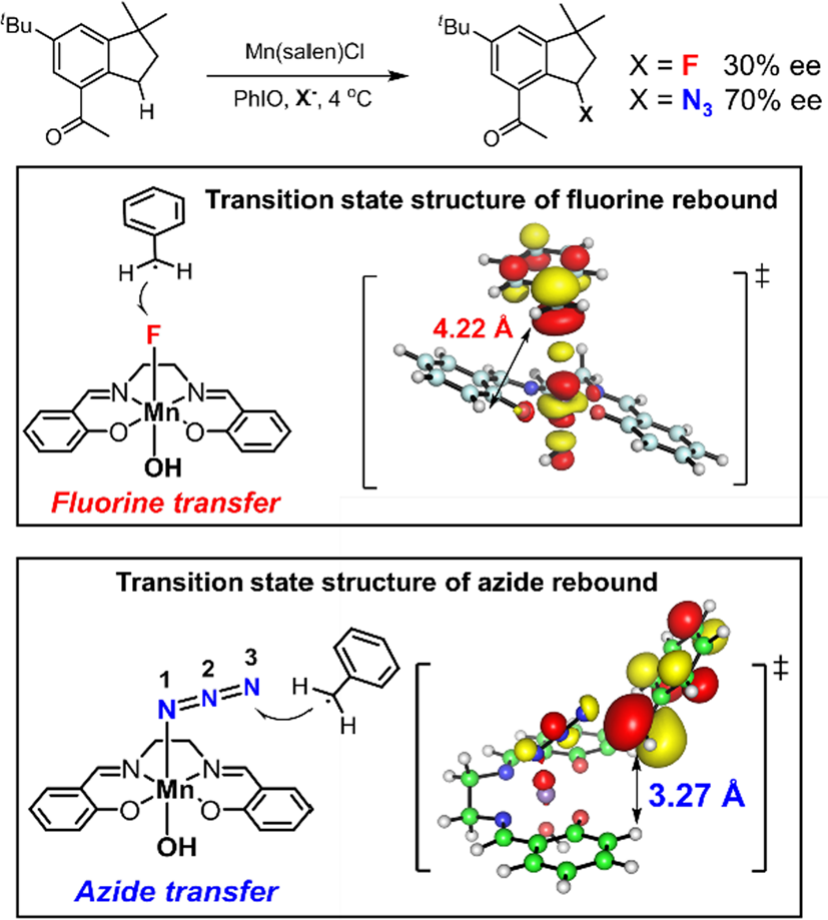
Comparison of the enantioselectivity between Mn-catalyzed C–H azidation and fluorination

The radical rebound approach is not only restricted to carbon–halogen bond formation reactions. Azide is another important functionality that has versatile applications in organic synthesis and biorthogonal chemistry [208, 209]. Azide transfer from metal-azide complexes to alkyl radicals is well known [210, 211]. We developed a manganese-catalyzed late-stage aliphatic C–H azidation reaction following the chemical logic of H abstraction/azide rebound (Fig. 28) [212]. Radical clock studies showed that the radical lifetime of this reaction was around 30 ns, significantly longer than what was observed for chlorination and fluorination. The azidation of celestolide with a chiral manganese salen catalyst afforded product in 70% ee, which is much higher than that of fluorination. DFT calculations showed that the azide transfer from Mn^IV^–N_3_ to an alkyl radical adopted a bent geometry for all reaction pathways because of the linear shape of azide, whereas the lowest-energy pathway of fluorine transfer showed a linear σ-geometry, pushing alkyl radicals away from the steric environment of the ligand, leading to low enantioselectivity (Fig. 29).

This series of unprecedented radical C–H functionalization reactions share common features and mechanistic foundations. They are all initiated by hydrogen abstraction via oxoMn^V^ intermediates, which give rise to the tunable regioselectivity that can surpass the restriction of innate reactivity of aliphatic C–H bonds. The oxygen rebound in these reactions are all suppressed due to the presence of strong anionic σ-donating ligands. Although more studies are needed to further elucidate the nature of non-oxygen rebounding intermediate, radical clock studies with norcarane showed long radical lifetimes (~2 ns for fluorination, ~10 ns for chlorination, and ~30 ns for azidation). The long radical lifetime seems to be crucial for the success of non-oxygenation reactivity, as changing the catalyst to more electron-withdrawing manganese porphyrins [such as Mn(TPFPP)Cl or Mn(TDCPP)Cl] or iron porphyrins suppresses the non-oxygenation reactivity and yields mainly oxygenation products. The stereoselectivity and the enantioselectivity of the reactions can be controlled by the catalyst through metal-mediated radical rebound. Such control of stereoselectivity is difficult to achieve with simple organic or inorganic radical trapping reagents.

The above examples show just a glimpse of the potential applications of radical rebound strategy in developing new radical organic transformations. Both the C–H activation and radical recombination approaches adopted by iron-containing oxygenases could likely be combined with current catalytic radical methodologies to afford new radical transformations. In one scenario, one can imagine that the carbon-centered radicals can be generated by the C–H activation via metal-oxo or similar metal-based hydrogen abstracting intermediates and subsequently be redirected to carbon–carbon or carbon-heteroatom bond formation via transition-metal mediated transformations. Such reactions would harness the controllable selectivity of metal-based hydrogen-abstracting intermediate in C–H activation step, which is hard to achieve with simple organic or inorganic radicals. In another case, carbon radicals could be generated with other radical initiation methods such as photoredox reactions, electrolysis, or other single-electron transfer (SET) processes, and later be captured by metal complexes [213–215]. The works of Kochi in 1970s have provided early examples of this type of reactions. He showed that various metal halide or pseudohalide complexes [i.e. CuBr_2_, Pb(OAc)_4_, etc.] were able to trap alkyl radicals generated by photolysis or thermolysis to afford halogenation or pseudohalogenation products [210, 211, 216]. Very recently, this area of research has seen dramatic progress mainly due to the development of photoredox catalysis. New methodologies have been developed for the construction of various carbon–carbon and carbon-heteroatom bonds via trapping the intermediate carbon radicals with transition-metal complexes especially nickel and copper [214, 217–223].

**Fig. 30:**
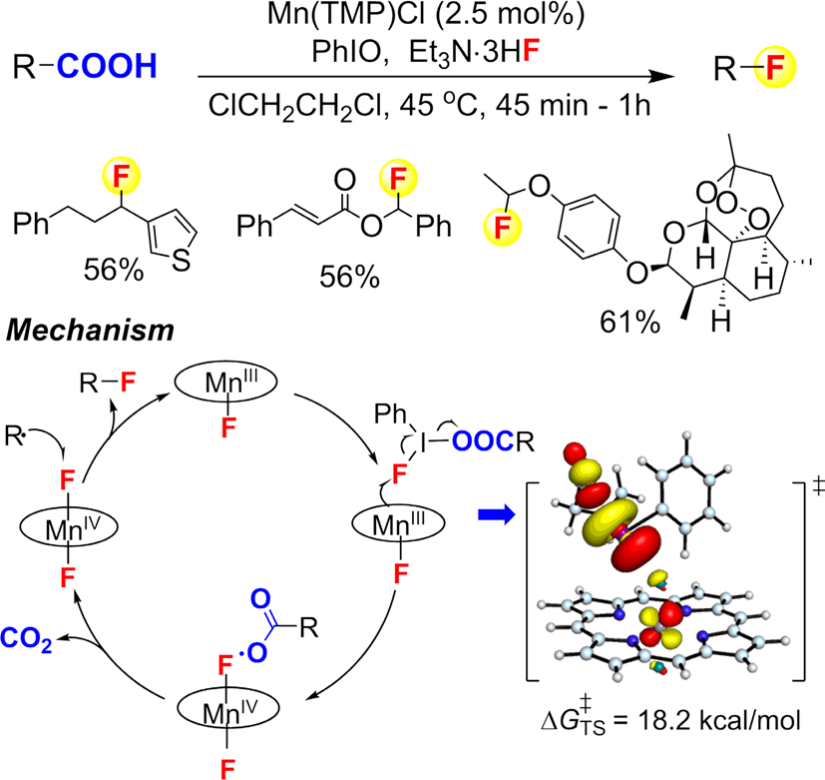
Mn-catalyzed decarboxylative fluorination

**Fig. 31:**
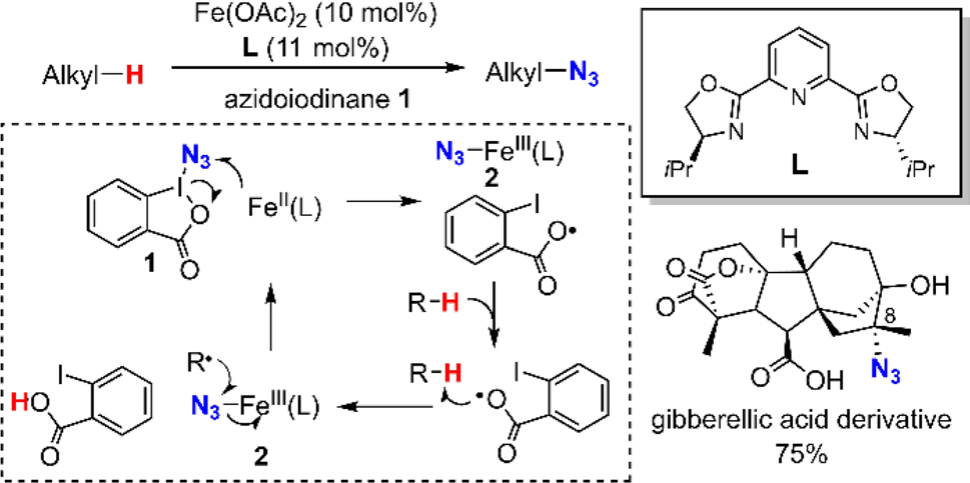
Fe-catalyzed aliphatic C–H azidation

Studies of Fe-containing oxygenases and related model compounds discussed above indicate that manganese and iron complexes also hold great premise for trapping the substrate radical intermediate, especially for the formation of carbon-halogen and carbon-pseudohalogen bonds. Following this design strategy, we recently developed the first decarboxylative fluorination reaction with fluoride ion (Fig. 30) [224]. In this reaction, the radical is generated via manganese-mediated oxidative decarboxylation. The generated radical is subsequently trapped by the Mn^IV^–F intermediate to afford fluorination product. Another compelling example was the aliphatic C–H azidation reaction reported by Hartwig and co-workers in 2015 [225, 226]. Mechanistic studies supported a reaction mechanism shown in Fig. 31. An initial one-electron oxidation between azidoiodinane **1** and iron(II) afforded a bezyloxyl radical and an azidoferric intermediate **2**. Benzyloxyl will abstract a hydrogen from the substrate and the substrate radical will be subsequently trapped by azidoferric intermediate **2** to afford azidation product. The reaction can functionalize complex molecules with multiple aliphatic C–H bonds with high regio- and stereo-selectivity. For example, a gibberellic acid derivative can be readily azidated giving a single diasterisomer with 75% yield. It is worth noting that in the absence of iron catalyst, azidoiodinane **1** can also azidate hydrocarbons with catalytic amount of radical initiators such as benzoyl peroxide with much lower stereoselectivity. This result highlighted the benefit of using metal complexes as radical trapping agents.

In addition to non-oxygen atom rebound, other reaction pathways adopted by iron-containing oxygenases are also highly valuable to the organic synthesis, such as alkane desaturation and radical cyclizations. There are relatively few studies on these reactions. The elucidation of their biochemical mechanisms and the design of synthetic model compounds to mimic the reactivities would definitely lead to new inventions in organic synthesis.

## Concluding remarks

It has been 40 years since our initial work on the radical rebound mechanism. The goals all along were to compare, contrast, and unify biocatalytic mechanisms with biomimetic systems. Iron-containing oxygenases continue to comprise a highly active research area with numerous new enzymes and numerous new reaction discoveries being made every year. What is fascinating about these enzymes is the diverse range of transformations they can catalyze. What is even more intriguing is the common mechanistic foundation underlying these various reactions: activation of oxygen to form high-valent iron–oxygen containing intermediates; abstraction of C–H bonds to yield radical intermediate; and the participation of the carbon-centered radicals in different reaction pathways giving rise to the diverse reactivities observed for iron-containing oxygenases. The study of the fundamental reactivities of these enzymes has inspired critical breakthroughs in synthetic organic chemistry, from early development of new molecular catalysts for alkane hydroxylations to the most recent progress in C–H halogenation reactions. We can expect iron-containing enzymes to continue powering the discoveries of new organic transformations because of the continuous discoveries and understanding of new reactivities of these enzymes as well as the recent renaissance of radical chemistry in organic synthesis.

## Abbreviations

cpd I: Compound I
cpd II: Compound II
TMP: Tetramesitylporphyrin
TPFPP: Tetrakis(pentafluorophenyl)porphyrin
TDCPP: Tetrakis(2,6-dichlorophenyl)porphyrin
4-TMPyP: Tetrakis(4-*N*-methylpyridyl)porphyrin
*α*KG: *α*-Ketoglutarate
